# Planar Asymmetries in the *C. elegans* Embryo Emerge by Differential Retention of aPARs at Cell-Cell Contacts

**DOI:** 10.3389/fcell.2019.00209

**Published:** 2019-09-27

**Authors:** Priyanka Dutta, Devang Odedra, Christian Pohl

**Affiliations:** Medical Faculty, Buchmann Institute for Molecular Life Sciences, Institute of Biochemistry II, Goethe University, Frankfurt, Germany

**Keywords:** planar polarity, asymmetry, cortical flow, PAR complex, CDC-42, morphogenesis, Wnt

## Abstract

Formation of the anteroposterior and dorsoventral body axis in *Caenorhabditis elegans* depends on cortical flows and advection of polarity determinants. The role of this patterning mechanism in tissue polarization after formation of cell-cell contacts is not fully understood. Here, we demonstrate that planar asymmetries are established during left-right symmetry breaking: Centripetal cortical flows asymmetrically and differentially advect anterior polarity determinants (aPARs) from contacts to the medial cortex, resulting in their unmixing from apical myosin. Contact localization and advection of PAR-6 requires balanced CDC-42 activation, while asymmetric retention and advection of PAR-3 can occur independently of PAR-6. Concurrent asymmetric retention of PAR-3, E-cadherin/HMR-1 and opposing retention of antagonistic CDC-42 and Wnt pathway components leads to planar asymmetries. The most obvious mark of planar asymmetry, retention of PAR-3 at a single cell-cell contact, is required for proper cytokinetic cell intercalation. Hence, our data uncover how planar polarity is established in a system without the canonical planar cell polarity pathway through planar asymmetric retention of aPARs.

## Introduction

Gradients in cortical tension can give rise to translocation of the contractile actomyosin network underlying the plasma membrane, a phenomenon called cortical flow (Chalut and Paluch, 2016). During animal development, cortical flow serves as a highly versatile biomechanical actuation system for cellular decision making due to differential spatiotemporal regulation and selective coupling to other cortically localized factors, e.g., polarity determinants, adhesion or signaling complexes. Polarized activation of cortical flow and transient, avidity-driven interactions have been shown to lead to advection of anterior polarity factors (aPARs) PAR-3, PAR-6, and PKC-3, thereby establishing the anteroposterior axis in *C. elegans* (Munro et al., 2004; Goehring et al., 2011; Dickinson et al., 2017; Wang et al., 2017; Mittasch et al., 2018). In addition to patterning the anteroposterior axis, where longitudinal cortical flow is required, dorsoventral and left/right (l/r) patterning in *C. elegans* require rotational cortical flow (Naganathan et al., 2014; Singh and Pohl, 2014; Pohl, 2015; Sugioka and Bowerman, 2018). Rotational flow emerges after formation of cell-cell contacts, where contact-dependent asymmetries determine cortical flow dynamics, which in turn determine spindle orientation through coupling to microtubule dynamics (Sugioka and Bowerman, 2018). These roles of cortical flow in patterning are orthologous in higher organisms, where they have been shown to drive decision making processes in development (Woolner and Papalopulu, 2012; Maître et al., 2016; Roubinet et al., 2017).

Despite the importance of cortical flows in polarized cell division, we know much less about the role of cortical flow during initiation of apicobasal polarity. In *C. elegans*, apicobasal polarity emerges during the second round of cell divisions. Here, the aPAR polarity determinants PAR-3, PAR-6, and PKC-3 that specified the anterior or somatic part of the embryo become restricted to the apical, contact free surfaces of blastomeres. For one of the apical polarity factors, PAR-6, an active process for its exclusion from basolateral cell-cell contacts has been identified (Anderson et al., 2008; Chan and Nance, 2013). While a RhoGAP, PAC-1, inactivates CDC-42 at basolateral contacts and thereby prevents recruitment of PAR-6 to these sites, at least two RhoGEFs, CGEF-1 and ECT-2, activate CDC-42, thereby counteracting PAC-1 (Chan and Nance, 2013). Whether and how these factors affect cortical flow at this stage and how other apical polarity determinants are regulated has not been analyzed so far. Moreover, *pac-1* loss-of-function embryos are viable despite transient mis-localization of PAR-6 (Anderson et al., 2008).

Previously, we demonstrated that shortly after the switch from anteroposterior to apicobasal polarization, an asymmetrically positioned midline forms in the *C. elegans* embryo through chiral morphogenesis, a rotational cell rearrangement with invariant directionality that is crucial for l/r symmetry breaking (Pohl and Bao, 2010). Chiral morphogenesis requires laterally asymmetric regulation of cortical contractility (Pohl and Bao, 2010) and is preceded by rotational cortical flows during division of the ectodermal blastomeres (Naganathan et al., 2014). The lateral asymmetry of cell movements and contacts strongly suggests that an unknown mechanism has to establish planar polarity at this stage. This elusive mechanism seems to utilize regulators involved in establishing apicobasal polarity, since chiral morphogenesis depends on CDC-42 (Pohl and Bao, 2010). Additionally, chiral morphogenesis also relies on non-canonical Wnt signaling (Pohl and Bao, 2010). The latter developmental signaling pathway has been shown to regulate embryonic spindle orientation (Schlesinger et al., 1999; Walston et al., 2004; Cabello et al., 2010; Sugioka and Bowerman, 2018) modulated by additional factors such as latrophilins (Langenhan et al., 2009) or syndecan (Dejima et al., 2014) and to determine cell fates in the early embryo (reviewed in Sawa and Korswagen, 2013) that seem to depend in part on Wnt-dependent induction of spindle asymmetry (Sugioka et al., 2011). Moreover, in *C. elegans* (Goldstein et al., 2006) as well as other systems (Habib et al., 2013), it has been shown that Wnt signaling can polarize isolated cells. In the *C. elegans* embryo, polarizing Wnt signaling emerges in the posterior blastomere, P1, and is then transduced from posterior cells to anterior cells by a relay mechanism that keeps re-orienting cells in the direction of the posterior polarizing center (Bischoff and Schnabel, 2006). This mechanism is most likely utilizing anteroposterior polarization of Wnt signaling components during mitosis as it has been shown that MOM-5/Frizzled is enriched at the posterior pole of cells before division in later embryogenesis (Park et al., 2004).

Hence, although Wnt signaling patterns the anteroposterior axis and is required for l/r symmetry breaking in *C. elegans*, roles of Wnt signaling in establishing planar cell polarity (PCP) have so far only been documented in neuronal morphogenesis. Here, Wnt signaling and the canonical PCP genes *fmi-1*/Flamingo, *prkl-1*/Prickle, *vang-1*/Van Gogh, *cdh-4*/Fat, *unc-44*/Diego regulate processes like fasciulation, neurite outgrowth, positioning and axon guidance (reviewed in Ackley, 2014). Importantly, neuro-morphogenesis as well as organogenesis in *C. elegans* results from interactions of individual cells with complex lineage trajectories and morphogenetic processes often occur in a local, piecemeal fashion (Bischoff and Schnabel, 2006; Rasmussen et al., 2008; Harrell and Goldstein, 2011; Pohl et al., 2012). Due to these developmental features, obvious planar polarized patterns based on polarity or PCP molecular markers have remained elusive for *C. elegans* embryogenesis.

In this study, we describe how planar polarized non-muscle myosin-aPAR domains form at the medial cortex at the time of l/r symmetry breaking in all the cells except P2. We find that non-muscle myosin-driven centripetal flow, which originates at cell-cell contacts, is at the core of this process. We characterize this process using particle image velocimetry (PIV), which reveals an anisotropy of flow emerging from anterior and posterior contacts. This in turn leads to the asymmetric positioning of aPAR domains through their advection from cell-cell contacts by centripetal cortical flow. We show that the embryonically required kinase GSK-3 (the glycogen synthase kinase 3β ortholog) is essential for release of non-muscle myosin from cell-cell contacts, which is crucial for flow-dependent advection of aPARs. In one blastomere, ABpl, planar asymmetries emerge at this stage, the most obvious one being PAR-3 retention at a single cell-cell contact. For emergence of planar asymmetry, active CDC-42 is required to recruit both PAR-3 and PAR-6 to cell-cell contacts, while the level of CDC-42 activation is critical only for PAR-6 advection. We quantitatively describe additional molecular asymmetries at cell-cell contacts including an inverse localization of CDC-42 GAPs and GEFs as well as activating and inhibitory Wnt signaling components. This type of planar asymmetry is later re-deployed during pre-morphogenetic development in posterior lineages. Thus, a balance between contact retention and release of non-muscle myosin and aPARs seems to determine the degree of advection, thereby controlling planar polarization of cell-cell contacts and the apical domain, constituting the first instance of obvious planar asymmetry in the early *C. elegans* embryo.

## Materials and Methods

### *C. elegans* Strain Maintenance

Strains were maintained on standard Nematode Growth Media (NGM) as previously described (Brenner, 1974) and were cultured at 20–25°C. Strain names and genotypes used in this study can be found in Supplementary Table S2.

### Mounting and Dissection of Embryos

Embryos were dissected from gravid hermaphrodites in M9 buffer on a cover slide. Embryos were selected at the 4-cell stage and mounted on a #1 coverslip (Corning, Lowell, MA, United States) along with a 1 μl suspension containing M9 and 20 μm diameter poly-styrene microspheres (Polyscience, Warrington, PA, United States). The preparation was then covered with another coverslip and sealed using vaseline. This sample preparation was imaged under the microscope usually starting from the ABa/ABp divisions.

### Live Cell Imaging

Appropriately staged embryos were imaged using a VisiScope spinning disk confocal microscope system (Visitron Systems, Puchheim, Germany) consisting of a Leica DMI6000B inverted microscope, a Yokogawa CSU X1 scan head, and a Hamamatsu ImagEM EM-CCD. Z-sectioning was performed with a Piezo-driven motorized stage (Applied Scientific Instrumentation, Eugene, OR, United States). All acquisitions were performed at 20–23°C using a Leica HC PL APO 63X/1.4-0.6 oil objective. For imaging/quantifying of anterior and posterior contacts of ABpl, we collected z-sections of 16 focal planes (0.5 μm apart) with 10 s intervals with a 488 and 561 nm laser at an exposure of 150 ms from the onset of ABa/ABp division, for a total duration of 10 min. While for PIV analysis, we collected 8 z-sections (0.5 μm apart) with 3 s intervals, again for a total duration of 10 min with the same laser settings as above. For imaging 12-cell stage embryos, we collected 26 z-sections (1 μm apart) with intervals of 1 min. For most imaging, laser intensities used were 5% for the 488 nm and 40 or 60% for the 561 nm laser (each 25 mW maximal output at the source).

### Replicates

The number of replicates per condition is mentioned for each condition or experiment individually. For each experiment shown, at least two biological replicates have been performed, for RNAi experiments at least three biological replicates have been performed and technical replicates from these have been pooled. Embryos with clear developmental problems (including shape or cell positioning defects), embryos undergoing cell cycle arrest and embryos that were improperly mounted have been excluded from our analysis.

### RNA Interference

RNAi experiments were performed by feeding as previously described (Kamath et al., 2003) with a few modifications in the amount of time that the animal is kept on the plate according to the lethality of the gene targeted (see below). RNAi feeding bacteria were grown overnight (around 16–18 h) in 1 ml Luria broth with ampicillin at a concentration of 100 μg/ml and 500 μl of this culture was used to inoculate 10 ml of LB ampicillin and grown at 37°C for 6–8 h. This culture was then centrifuged and resuspended in 300 μl of the same media, which was plated and kept for drying and induction on feeding plates (NGM agar containing 1 mM IPTG and 100 μg/ml ampicillin). Worms were kept on these feeding plates for the number of hours specified below and then dissected and mounted for imaging.

Weak RNAi perturbations of essential genes were performed by lowering the number of hours of feeding. The RNAi clone for *gsk-3* was availed from the Vidal library (Rual et al., 2004) and early L4 worms were kept on feeding plates for 24–36 h at 21–23°C and this was the temperature used throughout unless specified otherwise. *cdc-42, ect-2* feeding clones were also obtained from the Vidal library. For *cdc-42* RNAi, L4 or young adults were kept on feeding plates for 23–25 h. For *ect-2* RNAi, adults were kept on feeding plates for 12 h.

### Flow Velocity Analysis Using PIV

We used PIV to track NMY-2 and PAR-3 particles/foci in the apical cell cortex of ABpl and to measure velocity distributions with high spatial resolution. The imaging conditions we used for the PIV analysis were with high temporal resolution of 3 s intervals and with a high axial resolution of 0.5 μm spanning 8 z-stacks, enough to span the entire apical cortical section of ABpl. These z-stacks were projected using ImageJ and the image series was loaded into the PIVlab MATLAB algorithm (Thielicke and Stamhuis, 2014; pivlab.blogspot.de). In brief, a grid is drawn on each image. A fixed size window centered at each grid-point defines the region of interrogation. Fast Fourier transform is used to calculate the cross-correlations of this region with regions in the subsequent image. The PIV analysis was performed by using a 3-step multi pass, 64 × 64 pixel (9.152 × 9.152 μm), 32 × 32 pixel (4.576 × 4.576 μm) and the final interrogation window of 16 × 16 pixels with 50% overlap. Only the area within the apical cortex boundary of ABpl was taken for the analysis. The vector profiles generated using PIV gave us information about the direction and magnitude of the particle/foci movements. The vector fields across time were generated from each stage of a single embryo – accumulation, ventral movement and dissipation. The time-averaged vector fields from each stage were averaged for 3–4 embryos and are represented by wind rose plots. The MATLAB code is provided upon request. For statistical analysis of average velocities, a two-tailed *t*-test was used.

### Measurements

#### Fluorescence Intensities and Data Analysis

All quantifications of fluorescence intensities of proteins were performed on maximum intensity projections of apical cortical sections. For all measurements, background intensities were subtracted from the integrated intensity of the signals. For all measurements of fluorescence intensities at cell-cell contacts, the imaging conditions are mentioned above. We quantified the integrated intensity in circular ROIs of 0.327 μm at the cell-cell contacts of ABpl/ABpr using ImageJ. This diameter was small enough to span the entire width of the contact. For a single time point, we quantified 3 ROIs along the contact. The same ROI was used to measure the background intensity, which was subtracted from the signal intensity. For most of the measurements of cell-cell contact fluorescence intensities, we quantified 4 embryos. For medial cortex intensity measurements, we again quantified 3 circular ROIs of 0.327 μm for integrated intensity at every time point indicated. Most of the statistical analysis was performed using multiple *t*-test.

#### Center of Mass Calculation

We calculated the center of mass of the apical cortex of ABpl cell and the NMY-2 and PAR-6 domains using an ImageJ plugin. We manually traced the apical cortex boundary of the ABpl cell from z-projected stills to calculate the center of the mass of the apical cortex. We also manually traced the NMY-2 and PAR-6 domains similarly. We then subtracted the ABpl cell center of mass coordinates from the coordinates derived from center of mass of NMY-2 and PAR-6 domains to give us the center of mass of the domains relative to the center of mass of the cell.

#### Intercalation Lengths

We measured the intercalation lengths by measuring the distance from the apex of the intercalating lamellipodium to the edge of the lamellipodium from which the ABpl cell body starts.

## Results

### Advection and Unmixing of Apical Cortical Factors

**FIGURE 1:**
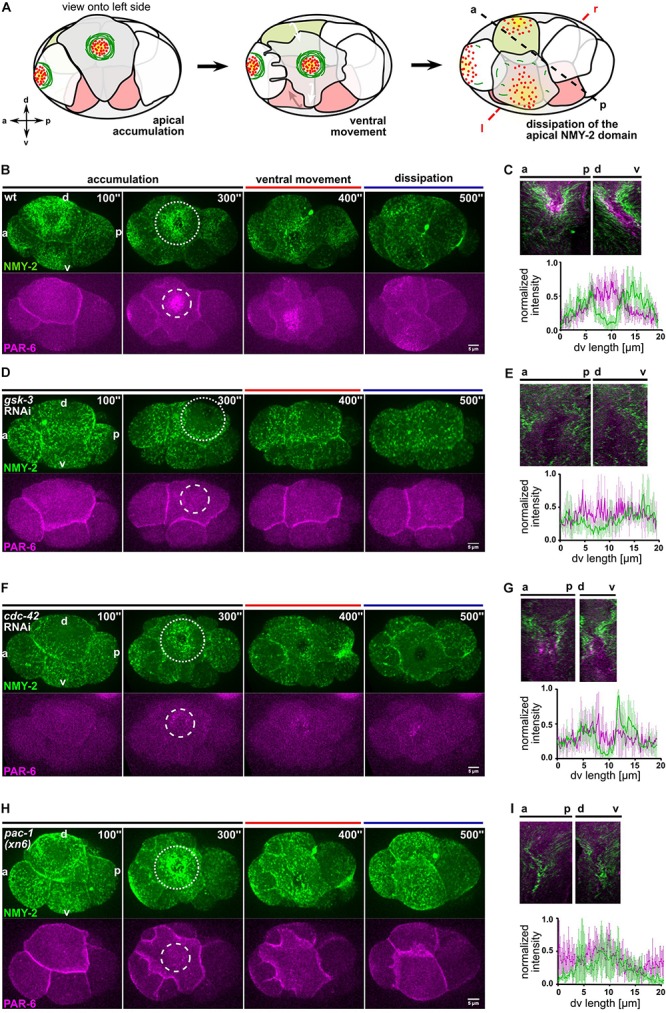
Centripetal actomyosin flow is essential for medial localization of PAR-6. **(A)** Schematic representation of the different stages of actomyosin flow on the left side of the embryo during chiral morphogenesis. Green depicts NMY-2, red PAR-6. **(B,D,F,H)** Representative time lapse images of projected apical sections of wt, *gsk-3* RNAi, *cdc-42* RNAi, and *pac-1(xn6)* embryos (shown is the left side) expressing NMY-2:GFP and mCherry:PAR-6 at the transition from 6- to 8-cell stage. Time is with respect to the completion of the ABp division. The axis directions are represented in the first timepoint. Dashed circles mark the NMY-2 and PAR-6 transiently stable apical structures, respectively. All images are representative of at least 10 embryos from 3 independent experiments. **(C,E,G,I)** Top: Kymographs of the apical cortex of ABpl along anteroposterior (a-p) and the dorsoventral (d-v) axis, showing the dynamics of cortical flow and localization of PAR-6 along time in wt **(C)**, *gsk-3* RNAi **(E)**, *cdc-42* RNAi **(G)**, and *pac-1(xn6)* **(I)** animals. Bottom: Normalized intensity plots of NMY-2:GFP and PAR-6:mCherry along d-v axis of ABpl apical cortex in wt **(C)** (*n* = 5), *gsk-3* RNAi **(E)** (*n* = 3), *cdc-42* RNAi **(G)** (*n* = 4), and *pac-1(xn6)* **(I)** animals (*n* = 4). Normalization was performed individually, for each genotype/RNAi. Scale bar = 5 μm.

*C. elegans* employs invariant l/r asymmetric contractility to establish an asymmetrically positioned midline through chiral morphogenesis (Pohl and Bao, 2010). This depends on the actin regulators Arp2/3 and CYK-1 (formin) as well as on non-canonical Wnt signaling (Pohl and Bao, 2010). Since previous studies imply roles of polarity factors in establishing apicobasal polarity at this stage (Nance et al., 2003; Anderson et al., 2008), we decided to explore how cortical contractile actomyosin flow impacts on them. We therefore quantitatively analyzed the dynamics of cortical and cell polarity factors (see section Materials and Methods; Zonies et al., 2010; Dickinson et al., 2013; Heppert et al., 2018) with high-resolution time-lapse microscopy. We observed that – except for P2 – all cells at this stage show centripetally directed myosin flow, emanating from apical cell-cell contacts and collecting at the apical center to form a stable ring-like structure for ABpl (Figures 1A,B, Supplementary Figures S1A,B and Supplementary Video S1) and crescent shaped structures for ABpr and MS (Supplementary Figures S1C–F and Supplementary Video S2). Centripetal flow seems equivalent to centripetal flow described during gastrulation (Pohl et al., 2012; Roh-Johnson et al., 2012). Concomitant with centripetal flow, PAR-6 accumulates at the center of the ring (Figure 1A, 200″–300″ time points; Figure 1B; Munro et al., 2004) and also adjacent to non-muscle myosin II, NMY-2, crescents (Supplementary Figures S1C,D). Hence, PAR-6 forms a compact, transiently stable, apical domain unlike the previously reported uniform localization (Anderson et al., 2008).

Since cortical NMY-2 and PAR-6 dynamics are also influenced by cell cycle progression and the cell movements of chiral morphogenesis (Pohl and Bao, 2010), we decided to analyze this developmental stage by dividing it into three phases, (I) initial apical accumulation of NMY-2 (0–300 s after completion of ABp cytokinesis), (II) ventral movement of ABpl (300–400 s), which is influenced by EMS cytokinesis and (III) dissipation of apical NMY-2 and PAR-6, which occurs during P2 division (400–500 s) (Figures 1A,B and Supplementary Table S1). Generally, we observed a dissipation of apical NMY-2 structures always around the onset of mitosis in embryonic blastomeres, at least until gastrulation. The accumulation of PAR-6 in the center of the NMY-2 ring occurs during centripetal flow (Figure 1C) and shows a time lag of 50 ± 7 s (*n* = 4) relative to maximal accumulation of NMY-2 at the medial cortex (Figure 1B and Supplementary Figure S1B). Based on previous modeling and experiments (Goehring et al., 2011; Mittasch et al., 2018) and since flow is fast enough (11 ± 2 μm/min, *n* = 4) as well as aPARs associate with the cortex sufficiently long enough (based on FRAP experiments, PAR-6 recovery half-time is 7 ± 2 s; *n* = 5), PAR-6 seems to be advected by centripetal cortical flow. Thus, while apical NMY-2 and aPARs accumulate together in the anterior half during polarization of the one-cell *C. elegans* embryo and stay segregated from PAR-2 only as long as flow persists (Cuenca et al., 2003; Munro et al., 2004), here, NMY-2 and PAR-6 unmix during centripetal flow and a medial PAR-6 domain persists for another 85 ± 9 s (*n* = 4) after NMY-2 centripetal flow has stopped (Figures 1B,C).

### Regulation of Centripetal Cortical Flow

Next, we asked whether centripetal flow is required for PAR-6 accumulation and performed a targeted screen (Supplementary Figure S2) that included factors known to affect rotational flow and chiral morphogenesis (Pohl and Bao, 2010; Naganathan et al., 2014; Singh and Pohl, 2014). For one of these factors, GSK-3, we observed a strong loss of cortical flow and a complete lack of cortical NMY-2 ring formation (Figure 1D, Supplementary Figure S3A and Supplementary Video S3). Importantly, cell division timing in these *gsk-3* RNAi embryos is by and large normal for AB blastomeres and no cortical flow phenotype or fate switch has been found for the first two divisions in *gsk-3* RNAi (Fievet et al., 2013; Du et al., 2014), arguing that loss of centripetal flow after GSK-3 depletion is a specific phenotype at this developmental stage. Consistent with advection being responsible for PAR-6 accumulation, *gsk-3* RNAi embryos show a complete lack of PAR-6 apical accumulation and precocious dissipation (Figures 1D,ESupplementary Video S3).

Furthermore, we wanted to test the role of the Rho GTPase CDC-42, which has been implicated in the localization of polarity factors at the apical cortex (Anderson et al., 2008; Chan and Nance, 2013). For this, we performed partial depletion of CDC-42 by short-term RNAi (24–28 h). Under these conditions, NMY-2 puncta are significantly reduced (Figure 1F), though this does not abrogate centripetal flow of NMY-2 and a stable ring configuration is formed (Supplementary Figure S3B and Supplementary Video S4). However, as a consequence of reduced NMY-2 puncta and apparently reduced myosin flow, PAR-6 accumulation is also substantially reduced and, in most cases, no discernible domain is visible (Figure 1F and Supplementary Figure S3B). Remarkably, lack of an apical PAR-6 domain leads to a collapse of the NMY-2 ring structure (in 22% of *cdc-42* RNAi embryos; *n* = 9), consistent with the idea that centripetal flow leads to a stable ring structure of NMY-2 due to unmixing and PAR-6 forming a stable, flow impenetrable domain after accumulation. Below, we demonstrate that this latter domain also contains all aPARs and most likely also other factors that become advected from cell-cell contacts. These findings are consistent with previous findings of CDC-42 being essential for apical localization of PAR-6 at this stage (Anderson et al., 2008).

In addition, a role for the RhoGTPase activating protein (RhoGAP) PAC-1 in driving the switch from anteroposterior to apicobasal polarization has been described previously (Anderson et al., 2008). While *pac-1(xn6)* embryos show normal centripetal NMY-2 flow (Figures 1H,I and Supplementary Video S5), they completely lack advection of PAR-6 (Supplementary Figure S3C). Hence, in accordance with the suggested role in apicobasal polarization (Chan and Nance, 2013), they show higher cell-cell contact levels of PAR-6 (Figure 1H and see below). Similar to CDC-42 depletion, in 22% of *pac-1(xn6)* embryos (*n* = 9), the NMY-2 ring structure collapses (Supplementary Video S5). Therefore, we conclude that while GSK-3 and CDC-42 are generally required for centripetal cortical flow at this stage, PAC-1 is specifically required for the release and advection of PAR-6 from cell-cell contacts, and both CDC-42 and PAC-1 are required to advect enough PAR-6 to generate a stable, flow-resistant medial apical PAR-6 cap.

### Anisotropic Centripetal Flow Generates Planar Polarized Cortical Domains

**FIGURE 2:**
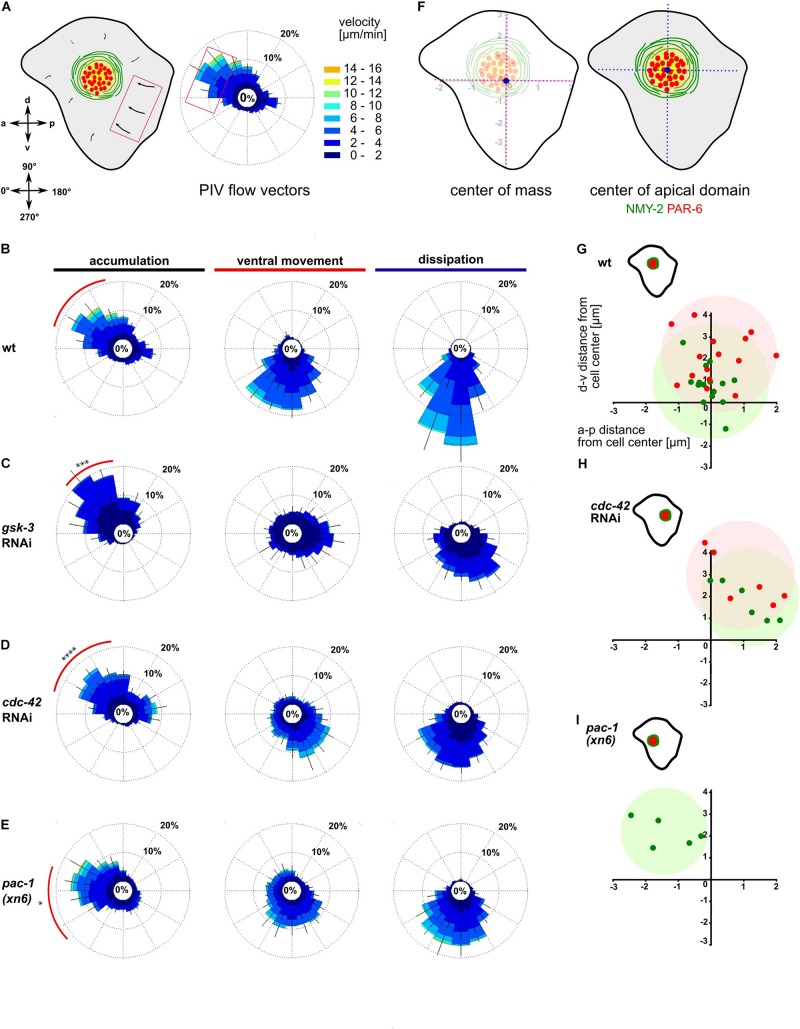
Anisotropic actomyosin flow generates planar polarized NMY-2-aPARs domains. **(A)** Left: Illustration of the ABpl apical cortex depicting the velocities of flow along a-p and d-v. Bottom: Axis direction correlated to angular coordinates. Middle: Wind rose plot depicting the direction and magnitude of cortical flow in the ABpl apical cortex generated by averaging velocities of NMY-2:GFP using PIV. Flow emerging from the posterior cell-cell contacts is represented in the anterior side of the plot and vice versa. Similarly, flow emerging from the ventral cell-cell contact is depicted in the dorsal side of the plot. Right: Color code for the magnitude of flow vectors. **(B–E)** Wind rose plots representing PIV analyses in wt **(B)**, *gsk-3* RNAi **(C)**, *cdc-42* RNAi **(D)**, and *pac-1(xn6)* **(E)** animals (*n* = 5). The red arc depicts the bias in a specific direction during the accumulation phase of more than 30% of the vectors with high magnitude. **(F)** Left: Illustration of center of mass of ABpl apical cortex (blue circle). Right: Center of mass of NMY-2 (green circle) and PAR-6 (red circle) in the apical cortex with respect to the center of mass of the ABpl apical cortex (blue circle). **(G–I)** Insets: Illustrations of the positioning of the NMY-2-aPAR cortical domain of the ABpl cortex. Plots show the positioning of NMY-2 (green) and PAR-6 (red) in wt **(G)**, *cdc-42* RNAi **(H)**, and *pac-1(xn6)* animals **(I)**; wt (*n* = 15) **(G)**, *cdc-42* RNAi (*n* = 6) **(H)**, and *pac-1(xn6)* (*n* = 5) **(I)**; data points show center of the respective fluorescence signal with respect to the center of mass of the ABpl cell cortex which is taken as coordinate (0,0). *P*-values: multiple *t*-test (^∗^*p* < 0.05, ^∗∗∗^*p* < 0.001, ^*⁣*⁣**^p < 0.0001).

Intriguingly, although centripetal cortical flow seems to symmetrically emerge from cell-cell contacts to the apical center in all embryonic cells at first glance, NMY-2 dissipative structures show a distinct, cell-specific anisotropic pattern (Supplementary Figures S1E,F and Supplementary Video S2). To ascertain cell-type specific structures and to answer the question as to which molecular asymmetries give rise to which cortical anisotropies, we decided to perform a detailed analysis of cortical flow in the cell driving this morphogenetic process, ABpl, through PIV (Figure 2A; Dutta et al., 2015). Quantification of flow velocities revealed that centripetal cortical flow is in fact anisotropic with flow emerging from posterior and ventral contacts having velocities ≤16 μm/min while cortical flow emerging from anterior and dorsal contacts only reaches ≤6 μm/min (Figure 2B). On average we find a centripetal flow velocity of 6 ± 2 μm/min in the direction with most flow vectors (Figure 2B, red arc). If we translate centripetal flow directions into angles (see wind rose plot description in Figure 2A), around 31% of the vectors are directing between 20 and 60°, exhibiting a bias toward the anterior direction (Figure 2B, red arc). PIV during ventral movement shows a strict ventral orientation of flow vectors which is also due to ventrally directed translocation of the cell itself (Figure 2B, middle). Subsequently, dissipation of cortical NMY-2 also occurs with a ventral oriented bias (Figure 2B, right).

We next sought to ask whether factors responsible for the regulation of cortical flow also control its asymmetry. We analyzed cortical dynamics of embryos partially depleted for GSK-3, CDC-42, and ECT-2 as well as *pac-1* mutants. Consistent with a strong loss of centripetal flow in *gsk-3* RNAi embryos, PIV of cortical NMY-2 shows significantly reduced average velocities (2.1 ± 0.4 μm/min, *p* = 0.0001) and the anisotropy being shifted so that most of the flow vectors direct in the range of 40–80°, with a stronger dorsal bias (Figure 2C, red arc). A partial lack of polarity was also evident during the ventral movement and dissipation phase (Figure 2C, middle and right). Depletion of CDC-42 caused average flow to be reduced (3.2 ± 0.8 μm/min, *p* = 0.0004) and the majority of flow vectors to be in the range of 20–90°, exhibiting a dorsal shift relative to wt (Figure 2D, red arc). In *pac-1(xn6)* embryos, the flow vectors as well as velocity increase in the 0–20° range, while the number of vectors and velocity decrease in the range of 40–60° (Figure 2E). Compared to wt having 25% of vectors with velocities of 4–6 μm/min in the range of 330–20°, flow vectors as well as velocity are increased in the 330–20° range with 44% having velocities of 8–10 μm/min, thereby exhibiting a strong anterior bias (Figure 2E, red arc). Overall, this leads to an increased average flow velocity in this particular direction (4.9 ± 0.7 μm/min, *p* = 0.01) compared to wt (3 ± 2 μm/min).

We next asked if this anisotropy in NMY-2 flow affects the apical, cell type-specific dissipative structures and to this end closely looked at the positioning of the NMY-2-aPAR domain in ABpl (Figure 2F). We observed that apical domain formation in wt embryos had a slight bias toward the anterior side (Figure 2G), in agreement with the polarization of flow (Figure 2B, red arc). Further, we wanted to explore if any change in anisotropy could lead to changes in the positioning of the apical structure. To this end, we analyzed the factors in which cortical flow anisotropy was altered. Partial depletion of CDC-42 leads to reduced anterior flow velocities and a dorsal flow bias, causing a posterior shift of the domain (Figure 2H). In *pac-1(xn6)*, the domain is shifted anteriorly (Figure 2I), again in concert with findings that flow velocities now have a stronger anterior bias with higher velocities. Partial depletion of ECT-2 leads to a posterior positioning of the domain similar to CDC-42 depletion (Supplementary Figure S4C and Supplementary Video S6). These results suggest that both the magnitude and direction of cortical flow velocities have roles in determining medial apical position of the domain.

### Differential Advection and Contact Retention of aPARs

**FIGURE 3:**
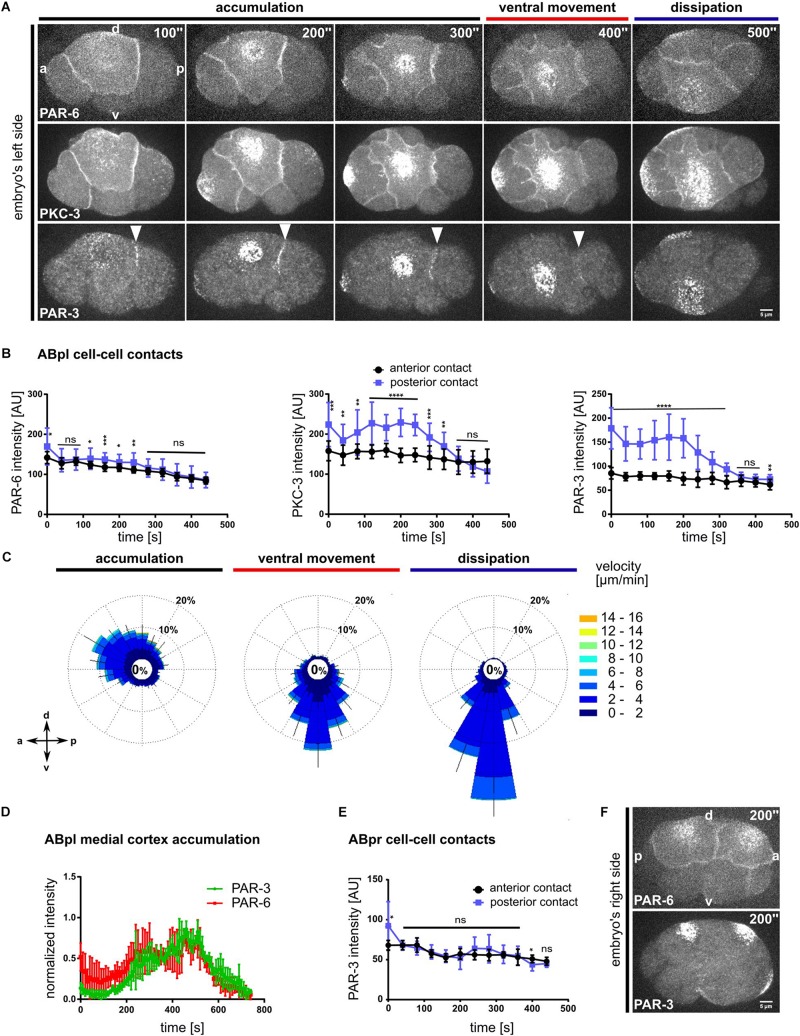
Advection and asymmetric contact retention of aPARs. **(A)** Representative time lapse images of apical cortical sections of embryos expressing mCherry:PAR-6 (top), GFP:PKC-3 (middle) and PAR-3:GFP (bottom). PAR-3 localization to the posterior contact (ABpl-P2) is marked by a white arrowhead. Time is with respect to the completion of the ABp division. All images are representative of at least 11 embryos from 3 independent experiments. **(B)** Quantification of mCherry:PAR-6 (*n* = 5), GFP:PKC-3 (*n* = 5) and PAR-3:GFP (*n* = 5) fluorescence intensities at the anterior (ABal-ABpl) and posterior (ABpl-P2) contact measured from apical cortical sections. Time is with respect to the completion of the ABp division. **(C)** Wind rose plots of PAR-3:GFP using PIV (*n* = 5). Right: Color code for the magnitude of flow vectors. **(D)** Normalized intensities of medial apical PAR-3 and PAR-6 fluorescence (*n* = 4). **(E)** Quantification of PAR-3:GFP fluorescence intensity at ABpr’s anterior (ABar-ABpr) and posterior (ABpr-P2) contact (*n* = 3). Time is with respect to the completion of ABp division. **(F)** Time lapse images of the right side of the embryo expressing mCherry:PAR-6 (top) and PAR-3:GFP (bottom). – All error bars indicate mean ± SD. *P*-values: multiple *t*-test (^∗^*p* < 0.05, ^∗∗^*p* < 0.01, ^∗∗∗^*p* < 0.001, ^*⁣*⁣**^p < 0.0001). Scale bar = 5 μm.

Recent reports about the differential functions of the different aPARs made us look more closely at their localization (Rodriguez et al., 2017; Wang et al., 2017). To this end we asked whether all aPARs show the same distribution and dynamics as PAR-6. The use of endogenously tagged transgenes and live imaging pinpoints this differential regulation: In contrast to previous data that relied mostly on immunostaining (Anderson et al., 2008; Chan and Nance, 2013), live cell imaging shows a substantial population of endogenously tagged PAR-6 being constitutively localized to apical cell-cell contacts (Figures 1, 3A and Supplementary Figure S5A). Comparing PAR-6 to the aPAR kinase, the atypical iota type protein kinase C, PKC-3, and to the other aPAR PDZ domain protein, PAR-3, we found that while all aPARs are advected by centripetal flow, PAR-6 and PKC-3 are also partially retained at all apical cell-cell contacts of somatic blastomeres in the embryo (Figure 3A, Supplementary Figure S5A and Supplementary Video S7). In contrast, PAR-3 is only retained at a single cell-cell contact, the contact between ABpl and the P2 blastomere on the left side of the embryo (Figure 3A, bottom, Supplementary Figure S5A, top and Supplementary Video S7). PAR-3 is present at this contact shortly after completion of ABp cytokinesis and remains localized at this contact until the start of ventral movement of ABpl (Figure 3B, right; Supplementary Video S7). Although PKC-3 and PAR-6 are retained at all somatic cell-cell contacts at this stage, quantitative analysis reveals that they nevertheless show an anteroposterior polarity with an enrichment at the posterior cell-cell contact of ABpl, which is weak for PAR-6 and stronger for PKC-3 (Figure 3B, left and middle). We speculate that posterior cell-cell contact localized aPARs must almost exclusively stem from the anterior cell (ABpl) since P2 only shows marginal levels of cortical aPARs much later when P2 divides into C and P3. Consistent with the idea that aPARs are advected by centripetal flow, we observe that PIV of PAR-3 in ABpl reveals the same directionality as NMY-2 flow, however, with slightly reduced average velocity (3.2 ± 0.5 μm/min versus 6 ± 2 μm/min; *p* < 0.0001; Figure 3C). Considering the obvious and measured (Dickinson et al., 2017) differences between PAR-3 and PAR-6 cortical assemblies, we could not detect a significant change in the kinetics of advection to the medial cortex (Figure 3D). However, this is due to the limitations of the PIV method used, which is efficient in detecting discrete rather than more continuous patterns such as PAR-6.

Moreover, no obvious cell-cell contact localization of PAR-3 can be observed on the right side of the embryo, in the fate-equivalent daughter of ABpl, ABpr (Figures 3E,F and Supplementary Video S8). Hence, at the stage of l/r symmetry breaking, PAR-3 seems to be part of a unique mechanism of cell-cell contact regulation that is laterally specific and polarized according to the anteroposterior axis, therefore representing a *bona fide* planar asymmetry.

### Regulation of aPAR Cell-Cell Contact Asymmetry

Given the importance of the above characterized regulators for centripetal cortical flow, we asked whether it is possible to modulate their levels to uncover specific functions in regulating cell-cell contact localization of NMY-2 and aPARs on one hand and advection on the other hand.

**FIGURE 4:**
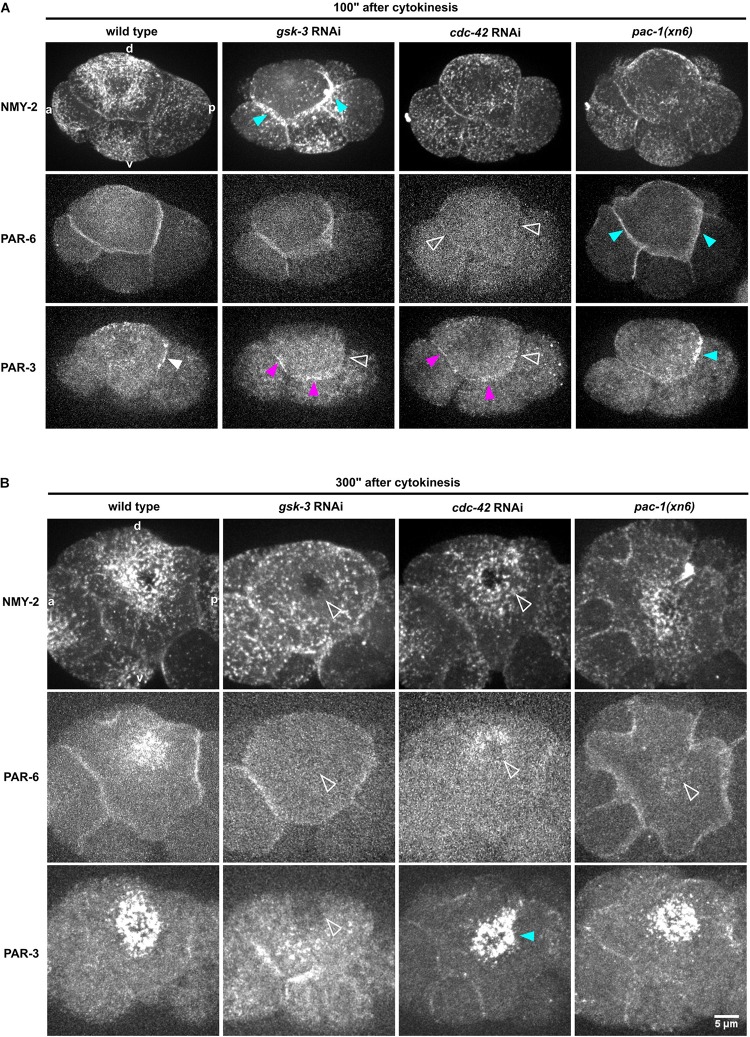
Regulation of NMY-2, PAR-6, and PAR-3 at the cell-cell contacts of ABpl. **(A)** Representative time lapse images of apical cortical sections of the left side of embryos expressing NMY-2:GFP (top), mCherry:PAR-6 (middle), and PAR-3:GFP (bottom) in wt and different RNAi/mutant backgrounds. Time is with respect to the completion of ABp division and just after advective flow starts. Effects of the RNAi conditions on PAR-3 localization. White arrowhead: wt localization; empty arrowhead: loss of cell-cell contact localization; blue arrowhead: increased localization; fuchsia: ectopic localization. All images are representative of at least 5 embryos from 3 independent experiments. **(B)** Representative time lapse images of enlarged apical cortical sections of ABpl expressing NMY-2:GFP (top), mCherry:PAR-6 (middle), and PAR-3:GFP (bottom) in wt and different RNAi/mutant backgrounds. Time is with respect to the completion of ABp division. Scale bar = 5 μm.

**FIGURE 5:**
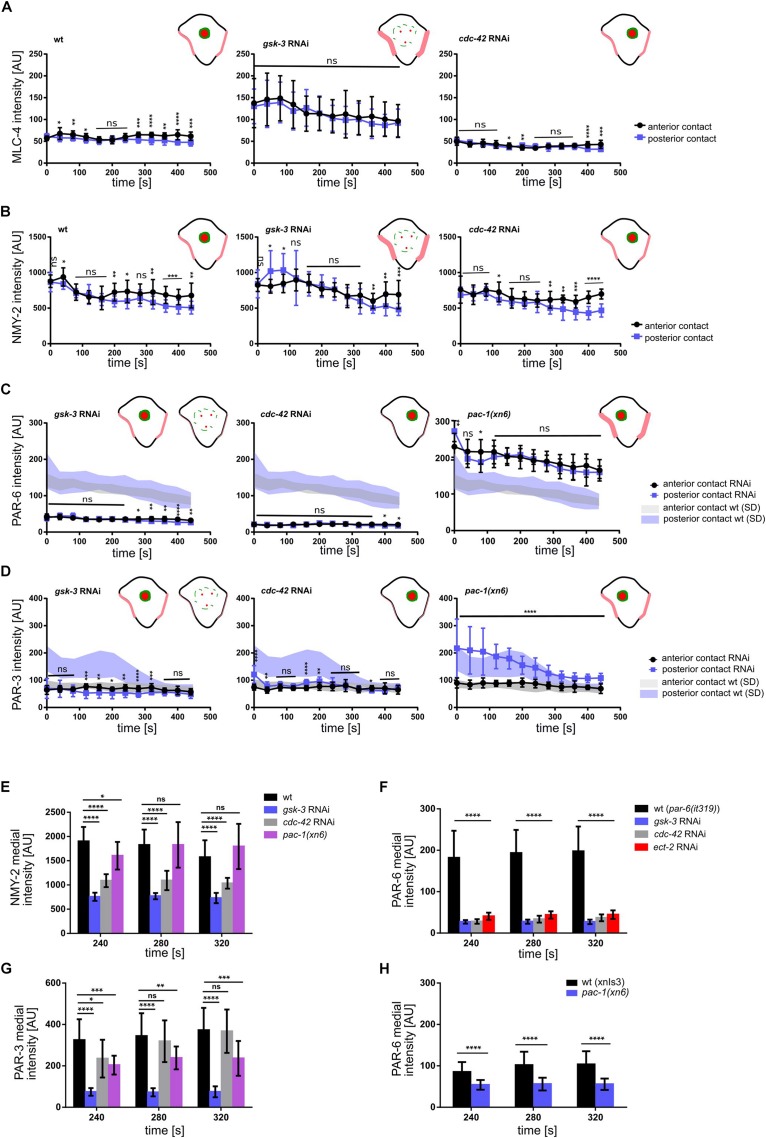
Regulation of NMY-2, PAR-6 and PAR-3 at the cell-cell contacts of ABpl. **(A–D)** Quantifications of mCherry:MLC-4 **(A)**, NMY-2:GFP **(B)**, mCherry:PAR-6 **(C)**, and PAR-3:GFP **(D)** at ABpl’s anterior (ABal-ABpl) and posterior (ABpl-P2) contact measured from apical cortical sections of the indicated backgrounds (*n* = 5). In **(C,D)**, the left schematic is to depict wt levels and the right schematic is to depict *gsk-3* RNAi levels**. (E–G)** Quantification of NMY-2:GFP **(E)**, mCherry:PAR-6 **(F)**, PAR-3:GFP **(G)** fluorescence intensity in the medial domain in RNAi conditions/mutant animals (*n* = 5). Time is with respect to the completion of ABp division. Error bars indicate mean ± SD. *P*-values: multiple *t*-test (^∗^*p* < 0.05, ^∗∗^*p* < 0.01, ^∗∗∗^*p* < 0.001, ^*⁣*⁣**^p < 0.0001).

To do so, we measured the levels of non-muscle myosin II heavy and light chains, NMY-2 and MLC-4, respectively, at the anterior and posterior cell-cell contacts of ABpl (the contacts ABal-ABpl, ABpl-P2, respectively) as well as at the medial cortex in wt and RNAi/mutant embryos to quantitatively characterize both contact localization and advection of the non-muscle myosin holo-complex. In addition, we performed the same measurements for PAR-3 and PAR-6. Measuring MLC-4 and NMY-2 at the anterior and posterior contact in wt embryos, we observed a slight anterior retention. Moreover, our quantifications revealed distinct roles for CDC-42, PAC-1, and ECT-2 in the cell-cell contact localization and advection of PAR proteins that are uncoupled from effects on centripetal cortical flow (Supplementary Figure S5B). Specifically, we found that CDC-42 is required for cell-cell contact localization of PAR-3 and PAR-6 (Figures 4A, 5C,D) but only for advection of PAR-6 and not PAR-3 (Figures 4B, 5F,G). Consistently, while centripetal flow is slightly reduced in *cdc-42* RNAi embryos (Figure 2D), we observed no significant change of PAR-3 advection to the medial cortical domain (Figures 4B, 5G). In addition and in line with previous findings that PAC-1 acts as a GAP for CDC-42 at this stage (Anderson et al., 2008; Chan and Nance, 2013), we found that *pac-1(xn6)* leads to an uncoupling of PAR-6 contact localization from advection: PAR-6 levels at cell-cell contacts are increased and medial cortex levels are highly reduced (Figures 4A,B, 5C,F). This is most likely due to higher levels of active CDC-42 at contacts, almost completely blocking advection. In this case, PAR-3 is still advected to the medial cortical domain, however, at reduced rates (Figures 4B, 5G). For us, the most likely interpretation for this is that increased levels of contact-localized PAR-6 will allow for formation of aPAR heterocomplexes and thereby to a slight increase of asymmetric, contact localized PAR-3. Moreover, consistent with ECT-2 serving as a GEF for CDC-42 at this stage (Chan and Nance, 2013), we find that it phenopcopies *cdc-42* RNAi for PAR-6 contact localization and advection (Figure 5F, Supplementary Figures S4A,B and Supplementary Video S6). However, *ect-2* RNAi does not lead to a loss of asymmetric PAR-3 contact localization (Supplementary Figure S4B), most likely due to ECT-2 acting redundantly with another CDC-42 GEF, CGEF-1 (Chan and Nance, 2013). Together, these results show that (1) flow can be uncoupled from advection for PAR-6: PAR-6 recruitment and advection are both blocked when CDC-42 is lost or rendered less active at contacts, while PAR-6 is locked at contacts when CDC-42 is overactivated; (2) PAR-3 advection occurs independently of CDC-42 and independently of PAR-6 advection and also under conditions of moderately reduced cortical flow; (3) asymmetric contact localization requires active CDC-42 but is not very sensitive to the level of CDC-42 activation (Supplementary Video S9). In addition, there is no reduction of overall PAR-6 (Supplementary Figure S6D) but only at the cortex and cell-cell contacts. Thus, de-regulation of CDC-42 at this morphogenetic stage does not simply induce pleiotropic phenotypes but allows to uncover differential regulation of aPARs.

In contrast to modulation of CDC-42 activity by RNAi, we observed that depletion of GSK-3 affects centripetal flow, cell-cell contact localization and advection of aPARs (Figures 4, 5). Upon GSK-3 depletion, NMY-2 levels are increased at cell-cell contacts (Figure 4A, blue arrows) and non-muscle myosin regulatory light chain, MLC-4, levels are increased almost threefold relative to wt levels (Figure 5A, middle). These data suggest that GSK-3 depletion leads to increased retention of the non-muscle myosin holo-complex, and, consequently, reduced apical flow. In contrast, CDC-42 depletion does not affect NMY-2 or MLC-4 levels at cell-cell contacts (Figures 4A, 5A,B), however, it leads to a similar degree of cortical flow reduction. This suggests that GSK-3 most likely acts upstream on pathways that translate progression of cell fates into executable morphogenetic programs through cortical factors.

### Cell-Cell Contact Asymmetry of Cortical Regulators, Signaling, and Cell Adhesion Molecules

**FIGURE 6:**
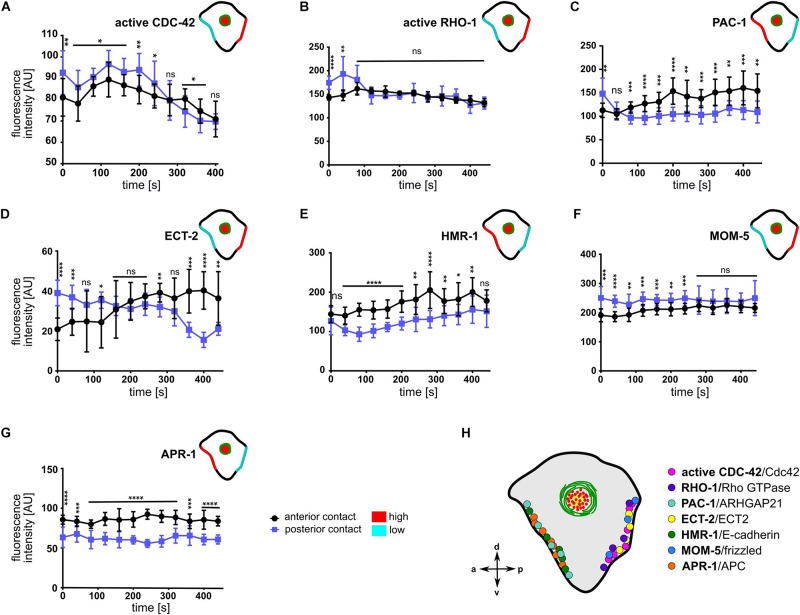
Asymmetric cell-cell contact localization of cortical regulators, signaling and cell adhesion molecules. **(A–G)** Quantification of fluorescence intensities of different transgenes at the anterior (ABar-ABpr) and posterior (ABpr-P2) contact of the ABpl apical cortex (*n* ≥ 3). Time is with respect to the completion of ABp division. Upper right inset: Illustration of the enrichment of the transgenes at the anterior and posterior contact; high: red, low: cyan. Error bars indicate mean ± SD. *P*-values: multiple *t*-test (^∗^*p* < 0.05, ^∗∗^*p* < 0.01, ^∗∗∗^*p* < 0.001, ^*⁣*⁣**^p < 0.0001). **(H)** Illustration of the dominant localization of the different factors at both cell-cell contacts.

The anisotropy in cortical flow (Figure 2), despite rather symmetric localization of NMY-2 and MLC-4 at cell-cell contacts (Figures 5A,B), suggested that Rho GTPases and their regulators might be playing a major role in creating cortical and contact asymmetries. To determine the possibility that CDC-42 might be asymmetric at cell-cell contacts, we used a sensor for activated CDC-42, a GFP-tagged CRIB/G-protein binding domain of WSP-1 (Kumfer et al., 2010). We observed a significant difference in the distribution of active CDC-42 in the anterior and posterior contacts (Figure 6A), with higher levels on the posterior contact. This is consistent with a positive role of CDC-42 in cortical flow (Fievet et al., 2013), since we observe directional flow with the highest velocities from posterior (Figure 2B). Contrary to previous reports (Anderson et al., 2008), however, we could not detect any cortically but solely cell-cell contact localized active CDC-42 (Supplementary Figure S6).

To examine the possibility that other Rho GTPases or their regulators might display an asymmetry at cell-cell contacts, we monitored a GFP-sensor for active RHO-1 (Figure 6B and Supplementary Figure S8; Tse et al., 2012). It showed an initial posteriorly biased asymmetry but became symmetrically localized at cell-cell contacts in later timepoints (Figure 6B). Next, we sought to examine the cell-cell contact distribution of the RhoGAP PAC-1. We detected an enrichment of PAC-1 at the anterior contact compared to the posterior contact (Figure 6C and Supplementary Figure S8). This is consistent with idea that PAC-1 curbs CDC-42 activity (Anderson et al., 2008; Chan and Nance, 2013) and with our data showing that CDC-42 is more active at the posterior cell-cell contact (Figure 6A). Moreover, quantifying a GFP-tagged ECT-2 transgene revealed an enrichment in the posterior contact compared to the anterior contact during accumulation phase (Figure 6D and Supplementary Figure S8). Thus, the GTPase cycle for CDC-42 seems to be spatially controlled, while the GAP shows an anterior bias, the GEF shows a posterior bias, which in itself would give rise to a posterior bias in recruitment of aPARs and stronger CDC-42-dependent centripetal cortical flows from the posterior. Moreover, this is consistent with the above data that advection of PAR-6 depends on balanced activation of CDC-42 (Figures 4, 5).

Since E-cadherin has been implicated in apicobasal polarization in several studies previously (Johnson et al., 1986; Nejsum and Nelson, 2009; Stephenson et al., 2010; Klompstra et al., 2015), we also decided to examine its localization at the contacts. Upon quantification, we found a significant anterior enrichment of HMR-1, the E-cadherin ortholog (Figure 6E and Supplementary Figure S8). This might relate to its role in translating specific contact cues into polarized recruitment of PAC-1 (Klompstra et al., 2015). Additionally, the Wnt pathway has been previously implicated in playing an essential role in chiral morphogenesis (Pohl and Bao, 2010) and MOM-5/Frizzled shows an anteroposterior asymmetric localization in cell divisions during later embryogenesis (Park et al., 2004). Therefore, we quantified MOM-5 levels at cell-cell contacts. Consistent with an anteroposterior polarization, we found that MOM-5 being asymmetrically localized at cell-cell contacts, with a posterior enrichment (Figure 6F and Supplementary Figure S8). Furthermore, APR-1, the ortholog of APC in *C. elegans*, is enriched at the anterior cell-cell contact (Figure 6G and Supplementary Figure S8) suggesting an opposing regulation of Wnt signaling at the anterior (Wnt low) versus posterior (Wnt high) cell-cell contact. These results are consistent with the anterior localization of APR-1 and posterior nuclear β-catenin localization in asymmetric seam cell divisions (Mizumoto and Sawa, 2007). Taken together, these results suggest that two opposing sets of protein complexes shape cell-cell contact asymmetry at this stage, one which is anteriorly and the other posteriorly enriched (Figure 6H).

### Morphogenetic Role of Planar Polarized PAR-3 Localization

**FIGURE 7:**
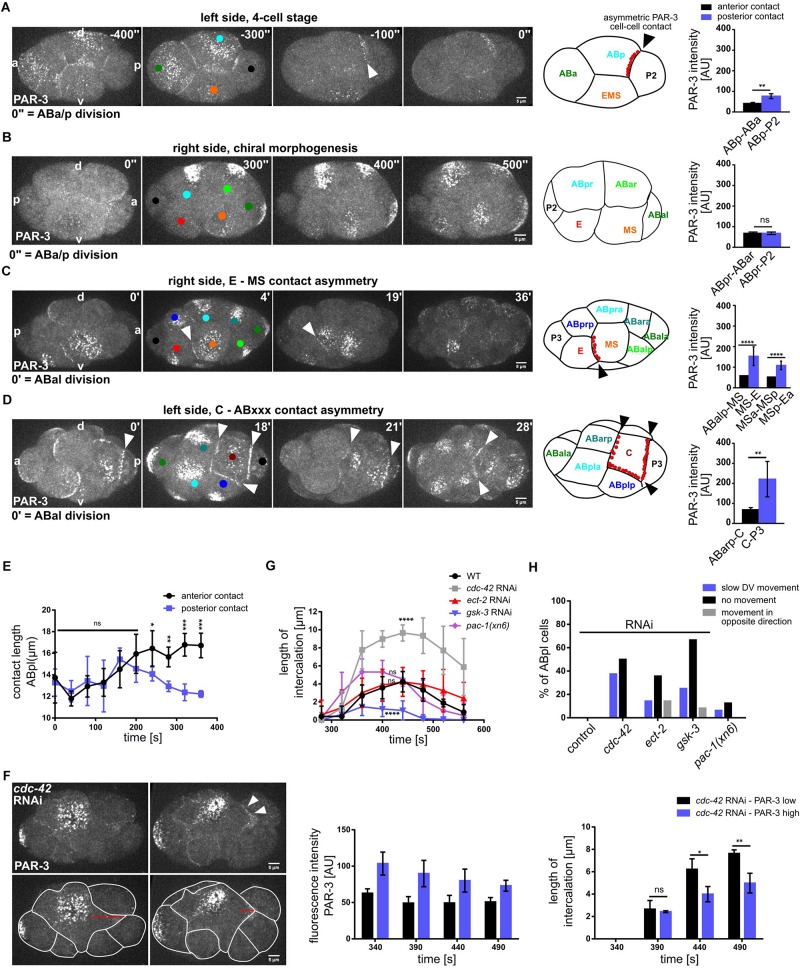
Morphogenetic role of cell-cell contact localized PAR-3 in posterior lineages. **(A–D)** Left: Representative time lapse images of cortical sections of embryos expressing PAR-3:GFP at 4 cell **(A)**, 6–8 cell **(B)**, 12-cell stage, right side **(C)**, 12-cell stage, left side **(D)**. White arrowheads point to cell-cell contact localized PAR-3. Cell identities are marked by colored circles, colors correspond to cell name colors in the embryo models (PAR-3 localization is marked in red). Right: Quantification of PAR-3:GFP fluorescence intensity at anterior and posterior contacts of ABp **(A)** (*n* = 2), ABpr **(B)**, MS and MSp **(C)** (*n* = 3), C **(D)** (*n* = 3), measured from apical sections. **(E)** Length of the anterior (ABa-ABp) and posterior contact (ABp-P2) at the ABpl apical cortex (*n* = 4). All images are representative of at least 5 embryos from 2 independent experiments. **(F)** Top left: Representative time lapse images of cortical sections of *cdc-42* RNAi embryos expressing PAR-3:GFP either at low level (upper left) or high level (upper right) at the posterior contact. Bottom left: Intercalation lengths of ABpl during the P2 division for both the above mentioned conditions. Middle: Quantifications of PAR-3:GFP fluorescence intensity in *cdc-42* RNAi embryos with low PAR-3 levels (black) and high PAR-3 levels (violet) at the posterior cell-cell contact. Right: Length of the intercalation of ABpl into the P2 furrow for the embryos quantified in the middle panel (*n* = 3). Time is with respect to the completion of ABp division. Error bars indicate mean ± SD. *P*-values: multiple *t*-test (^∗^*p* < 0.05, ^∗∗^*p* < 0.01, ^∗∗∗^*p* < 0.001, ^****^p < 0.0001. **(G)** Changes of the length of the intercalation of ABpl during the P2 division for different RNAi and mutant conditions over time (*n* = 8). Time is with respect to the completion of ABp division. **(H)** Quantification of different effects of the RNAi/mutant conditions on the ventral movement of ABpl during chiral morphogenesis (*n* ≥ 12). Scale bar = 5 μm.

The localization of PAR-3 to a single cell-cell contact during chiral morphogenesis prompted us to analyze PAR-3 localization during subsequent stages of embryogenesis. Intriguingly, we found that PAR-3 shows highly lineage-specific asymmetric cell-cell contact localization (Figures 7A–D). Specifically, the first contact that shows clear PAR-3 localization is the contact between ABp and P2 (Figure 7A). This localization is not restricted to one side of the embryo and thus also not planar polarized, however, it occurs prior to establishment of l/r asymmetry. As mentioned above, localization to the ABpl-P2 and later to the ABpl-C cell-cell contact is unique and not mirrored on the right side of the embryo by ABpr (Figure 7B). Subsequently, PAR-3 localizes to the MS-E cell-cell contact (Figure 7C and Supplementary Video S10). This boundary between mesoderm and endoderm is maintained after the division of MS (Figure 7C). At the same developmental stage, on the other side of the embryo, PAR-3 localization between ABpl and C is lost during ABxx cell divisions but is rapidly re-established after completion of the divisions (Figure 7D). Slightly earlier, PAR-3 starts to accumulate at the C-P3 cell-cell contact. Like this, the C blastomere is almost completely encased by PAR-3-containing cell-cell contacts (Figure 7D and Supplementary Video S11). Collectively, this shows that PAR-3 marks cell-cell contacts to generate a planar polarized pattern in the embryo that demarcates specific posterior lineages, E, C, and P.

To better understand the roles for PAR-3 contact localization, we decided to study the ABpl-P2 cell-cell contact in more detail. Unlike the other blastomeres in the embryo at this stage, the cell-cell contacts of ABpl undergo a highly asymmetric development during the ventral movement of the cell, the anterior contact expands while the posterior shrinks (Figure 7E). We therefore asked how PAR-3 localization affects contact dynamics. When comparing wt and *cdc-42* RNAi embryos, we found that the contact between ABpl and P2/C shows very different dynamics (Figure 7F, left). Unlike wt embryos, ABpl fully intercalates into the furrow of the dividing P2 cell in *cdc-42* RNAi embryos (Figure 7G and Supplementary Video S4). Due to the variability in depletion by RNAi, we analyzed *cdc-42* RNAi embryos with strongly reduced ABpl-P2 cell-cell contact localized PAR-3 and compared these to *cdc-42* RNAi embryos with moderate reduction of PAR-3 (Figure 7F, middle). Consistent with the idea that PAR-3 contributes to separation of anterior from posterior cells, we found that embryos with strong PAR-3 reduction also show significantly increased erroneous furrow intercalation (Figure 7F, right). When averaging over several embryos, *cdc-42* RNAi embryos show a significant increase in ABpl-P2 furrow contact length (Figure 7G) while this is not significant for *ect-2* RNAi and *pac-1(xn6)* embryos (Figure 7G). We suggest that this is due to decreasing impact of these backgrounds on asymmetric PAR-3 cell-cell contact localization in the order *cdc-42*, *ect-2*, *pac-1* RNAi (Figure 5D and Supplementary Figure S4B). Previously, using laser irradiation-inflicted cell cycle delays, we could show that intercalation of ABpl into the EMS furrow is essential for proper movement of ABpl and generation of a tilted midline (Pohl and Bao, 2010). Consistently, ABpl fails to move ventrally when it retracts from the EMS furrow in all *gsk-3* RNAi embryos (Supplementary Video S3) as well as *ect-2* RNAi embryos (9/14). Taken together, problems in chiral morphogenesis (where the ABpl cell does not migrate ventrally), have different reasons (Figure 7H): In *cdc-42* RNAi embryos, this is mostly due to slowing down of ventral movement by erroneous intercalation of ABpl into the P2 furrow, while in *ect-2* RNAi this is due to most RNAi embryos (10/14), showing a retraction of the ventral ABpl lamellipodium that intercalates into the EMS furrow. Similarly, in *gsk-3* RNAi embryos chiral morphogenesis fails due to lack or instability, respectively, of intercalation of ABpl into the EMS furrow (Figure 7H).

Finally, based on these findings, we decided to investigate whether aside from regulating cytokinetic cell intercalation at the ABpl-P2/C contact, cell-cell contact localized PAR-3 also regulates cytokinetic cell intercalation during later stages. To do so, we focused on the MS-E contact that also shows a very clear PAR-3 enrichment (Figure 7C). Here, during cytokinesis of the MS blastomere, neighboring AB-derived blastomeres readily intercalate into the MS furrow while E does not (Supplementary Figure S7, white outlines). Moreover, we also observe that a large fraction of cortical PAR-3 accumulates in the midbody in the subsequent division of the E blastomere (Supplementary Figure S7; arrowhead) and the remaining apical PAR-3 stays associated with the Ea-Ep cell-cell contact. This is only observed for the E blastomere and not for any other blastomere, arguing that clearing of apical PAR-3 through this mechanism might help to prepare Ea and Ep for gastrulation where they are covered by neighboring cells (Nance et al., 2003). Thus, a main function of cell-cell contact localized PAR-3 seems to regulate cytokinetic cell-cell intercalation which can give rise to substantial cell rearrangements in a structure that is only composed of a few cells.

## Discussion

### Early Establishment of Planar Polarity in *C. elegans*

More than 20 years ago, the concept was established that blastomeres in *C. elegans* are specified by a process of stepwise, binary diversification involving the Wnt pathway genes *lit-1*/NLK and *pop-1*/TCF/LEF (Kaletta et al., 1997; Lin et al., 1998). Subsequently, it was shown that the core of this specification system is a relay of Wnt-dependent spindle-polarizing information that originates in the germline blastomere P1 and is maintained in its descendants (Bischoff and Schnabel, 2006). Since the *C. elegans* embryo can be considered a squamous-like epithelium, this specification system will most likely require planar polarized domains to prevent cell-cell mixing at cell fate boundaries and during cell division, particularly since cell divisions usually generate anteroposteriorly staggered configurations. Here, we demonstrate that after the transition from anteroposteriorly polarized blastomeres in the 2-cell to apicobasally polarized blastomeres in the 4-cell embryo, specific blastomeres also become polarized in the plane of the embryonic epithelium. We show that planar asymmetries are established through deployment of the same machinery that patterns anteroposterior and apicobasal polarity, cortical contractile actomyosin flow together with anterior/apical polarity determinants, most importantly, PAR-3. It seems plausible to speculate that the most likely physiological function of transiently stable apical domains is to provide cells with the ability to undergo a shape transition after exit from mitosis. At this early stage, blastomeres neither have developed canonical cell-cell junctions (whose remodeling would lead to new configurations of cells) nor do they form bona fide protrusions at this stage that would allow them to migrate directionally. In other words, we consider apical domains at this stage the main cellular mechanism to control cell shape during cell-cell re-arrangements outside mitosis (where this is regulated by spindle orientation).

In addition to the first cell with obvious planar asymmetries, ABpl, which drives l/r axis formation, planar polarization of cells is restricted to cell-cell contacts with posterior lineages that need to give rise to (mostly) clonal tissues, germline (P2 and P3), endoderm (E and Ex), and laterally symmetric body muscle (C and Cx). We present evidence that planar polarized landmarks on these lineages seem to help in preventing cell-cell intercalation during division of these lineages (Figure 7 and Supplementary Figure S7). In many embryos, regulated, cell division-mediated intercalations contribute to cell movements and patterning during early development, including the Drosophila (Founounou et al., 2013; Guillot and Lecuit, 2013; Herszterg et al., 2013), the chick (Firmino et al., 2016), and the Xenopus embryo (Hatte et al., 2014). This is due to the fact that cytokinesis has to adapt to the multicellular context, where the dividing cell biomechanically signals the need for adhesion remodeling to the neighboring cells (Herszterg et al., 2014). The situation in *C. elegans* is slightly different than in those organisms since furrowing is asymmetric and progresses from apical to basal, where the midbody is then localized, while in many other organisms, midbodies of embryonic epithelia end up on the apical side (Herszterg et al., 2014). This difference is most likely due (1) to the lack of polarized apical junctions in *C. elegans* that can serve as a mount for the actin cytoskeleton in other organisms, and (2) since the early *C. elegans* embryo is topologically different from other embryonic epithelia, consisting of a small number of squamous-like blastomeres where cell-cell contact rearrangements appear more similar to those in early embryos of other holoblastically cleaving species like mouse or human. Interestingly, although a stochastic process, lineage segregation depends on the inheritance of the apical domain in the mouse embryo (Maître et al., 2016; Korotkevich et al., 2017), highlighting a conserved function of apical polarity determinants in cell fate specification.

Our data support parts of our earlier model on the integration of mechanisms leading to a continuum of axial patterning in *C. elegans* (Pohl, 2015): It was previously shown that Wnt signaling, known to regulate chiral morphogenesis, is also required for chiral, counter-rotating flow during skewing of the ABa/ABp division (Naganathan et al., 2014). Based on these findings, we propose that directional cortical flow during cytokinesis of ABp might bias the distribution of Wnt pathway components such as MOM-5/Frizzled to become enriched on the ABpl/P2 interface (Figure 6F). This in turn might lead to anterior enrichment of antagonistically acting factors like APR-1/APC (Figure 6G; Mizumoto and Sawa, 2007). It seems plausible to speculate that factors acting downstream on cortical flow and aPAR advection/retention such as CDC-42 and its regulatory GAPs and GEFs receive instructive inputs from asymmetrically localized Wnt signaling as Wnt signaling has been shown to polarize other cytoskeletal structures such as the spindle (Goldstein et al., 2006; Sugioka et al., 2011; Sugioka and Bowerman, 2018). Thus, the intrinsic chirality of actomyosin dynamics during cytokinesis together with the impact of polarized Wnt signaling might constitute the main driver of axial patterning coordination once cell-cell contacts exist in the embryo (Sugioka and Bowerman, 2018).

### Role of Rho GTPases and Their Regulators in Planar Asymmetry

**FIGURE 8:**
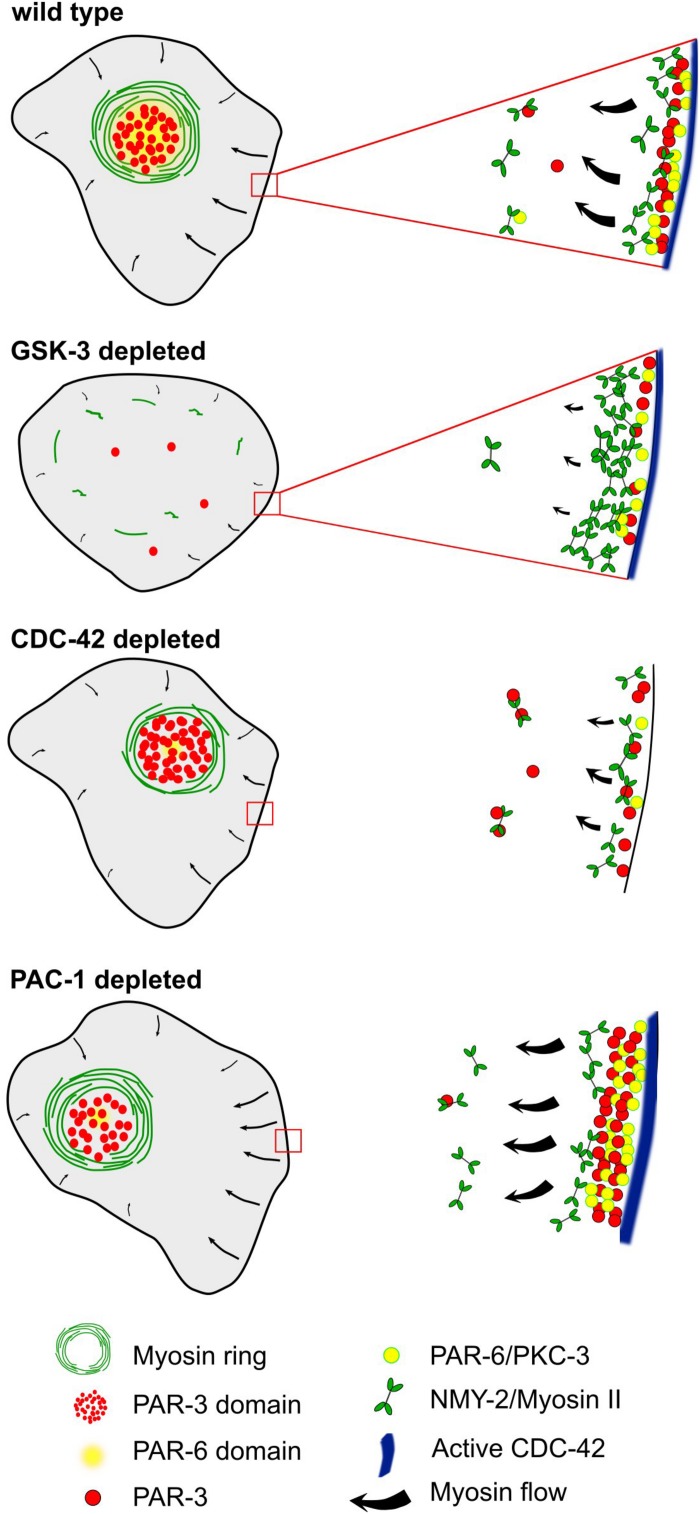
A model for the emergence of planar polarized myosin-aPARs medial domains. Centripetal cortical flow in wt, emerging from cell-cell contacts, leads to advection of apical polarity determinants. Differential spatial regulation at contacts induces asymmetric retention of a subset of them, most prominently PAR-3 and PKC-3. When deleting GSK-3, NMY-2 is retained at cell-cell contacts, resulting in strongly reduced centripetal cortical flow. Depletion of CDC-42 reduces centripetal flow, strongly reduces recruitment of PAR-6 and renders cell-cell contacts unable to retain PAR-3. In contrast, reducing the activity of the RhoGAP, PAC-1, leads to increased retention of PAR-6 at cell-cell contacts, resulting in reduced advection, while not affecting PAR-3.

Previously, a role of cortical flow in controlling clustering of aPARs has been described for the polarization of the anteroposterior axis (Wang et al., 2017). Here, cortical flow enables clustering of PAR-3 as a response to cortical actomyosin contractility-generated tension. Moreover, reduced activity of CDC-42 allows the other aPARs, PAR-6, and PKC-3, to associate with PAR-3 clusters, while increased CDC-42 activity leads to a more diffuse cortical localization of PAR-3 and dissociation of aPAR co-clusters (Wang et al., 2017). Vice versa, PAR-3 clustering has been shown to be required for effective advection (Dickinson et al., 2017). Moreover, consistent with CDC-42 activity shaping aPAR complexes, formation of clustered versus diffuse aPAR complexes during anteroposterior polarization also depends on an inverse activity state of PKC-3 (Rodriguez et al., 2017), giving rise to clustered PAR-3-PAR-6-PKC-3^*inactive*^ (corresponding to the co-clustered aPAR complex with CDC-42low; Wang et al., 2017) and diffuse CDC-42-PAR-6-PKC-3^*active*^ (corresponding to aPAR co-cluster dissociation or CDC-42^*high*^, Wang et al., 2017). Although a different developmental stage, our data strongly support this type of aPAR complex regulation (Figure 8): In the first cell with planar asymmetric PAR-3 at cell-cell contacts, ABpl, we find that CDC-42 activity is presumably high in the posterior cell-cell contact due to the CDC-42-inactivating GAP, PAC-1, showing the reciprocal planar asymmetry of PAR-3 (Figures 6A,C). Notably, also the CDC-42 GEF, ECT-2, and active RHO-1 are initially enriched posteriorly (Figure 6B). Consistent with the findings during anteroposterior polarization, this would lead to dissociation of aPAR co-clusters at the posterior cell-cell contact, where more active CDC-42 is localized (Figure 6A). This also explains, why not only PAR-3 but also PAR-6 and PKC-3 show planar asymmetry, although not as pronounced as PAR-3 (Figure 3B). Accordingly, we find that PAR-3 is more readily advected and lost from ABpl’s posterior contact when CDC-42 levels are down-regulated (Figure 5G). Therefore, it seems plausible that when cortical flow emerges in vicinity of cell-cell contacts (where CDC-42 is no longer detectable), centripetal cortical flow might again trigger aPAR co-clusters that we find to be co-advected to the medial cortex (Figure 3D). However, unlike during the anteroposterior polarization, centripetal cortical flow is not able to advect all PAR-6 and PKC-3 from cell-cell contacts, which can be attributed to the interaction with contact-localized CDC-42 and interaction with cell-cell adhesion complexes that obviously did not exist in the one-cell stage. Remarkably, PAR-6’s interactions with cell-cell contact-localized factors seems to be specifically regulated by PAC-1, which, when mutated leads to loss of PAR-6 advection by centripetal flow, also from contacts with lower levels of PAC-1 (Figure 1H). These findings are mostly consistent with previous data (Klompstra et al., 2015), showing a multi-component protein complex scaffolded by E-cadherin recruiting PAC-1 to cell-cell contacts. Interestingly, we find that ABpl’s anterior cell-cell contact shows significantly higher HMR-1/E-cadherin levels than the posterior, which can explain the observed anterior PAC-1 enrichment (Figure 6E). We can only speculate that this asymmetric localization of PAC-1 might also contribute to enhanced levels of cell-cell contact F-actin and reinforce recruitment of cell-cell adhesion proteins as described for late stages of embryonic morphogenesis (Zilberman et al., 2017).

### Similarity and Difference to Other Forms of Planar Polarity

During gastrulation in Drosophila, correct anteroposterior patterning of the extending germband requires planar asymmetry of non-muscle myosin II localizing to anteroposterior cell-cell contacts while Bazooka/PAR-3 localizes to dorsoventral contacts (Zallen and Wieschaus, 2004). Although PAR-3 localizes to the posterior cell-cell contacts in ABpl, the lack of hexagonal epithelia that are mostly controlled by junction mechanics-dependent neighbor exchanges, makes it difficult to compare the role of planar polarized cell-cell contacts in *C. elegans* to those in the early fly embryo. However, molecularly, there seem to be several similarities. For instance, it has been shown that for sensory organ precursor cells (SOPs) in the notum epithelium, PCP depends on the canonical PCP pathway involving, among others, *fz/frizzled*, *dsh/disheveled, Vang/Van Gogh*, and *fmi/Flamingo* (reviewed in Yang and Mlodzik, 2015). In the absence of PCP, SOPs divide with properly segregated antagonistic polarity domains (aPARs versus Pins/Numb), however, randomly with respect to the epithelial plane (Besson et al., 2015). Interestingly, aPAR domains already polarize before mitosis in dependence on Wnt/PCP. Therefore, similar to our data on the emergence of planar asymmetries of aPARs in the early *C. elegans* embryo, there also seem to be Wnt/PCP-dependent mechanisms that operate outside of their known roles in mitosis and spindle orientation (Yang and Mlodzik, 2015). Moreover, in the Drosophila ommatidial epithelium, PCP controls the unilateral localization of Bazooka/PAR-3, independently of Par-6 (Aigouy and Le Bivic, 2016), again highly similar to the pronounced asymmetry of PAR-3 at posterior cell-cell contacts in *C. elegans* that does not in all cases require proper regulation of PAR-6, for instance in *pac-1(xn6)* (Figures 4A, 5D).

In vertebrates, Par3’s role in planar polarity has been reported to be either uncoupled from or coupled to its role in apicobasal polarity, depending on the context. During mouse inner ear development, Par3 is asymmetrically localized in dependence on canonical PCP and Rac signaling but independently of Par6 and aPKC, moreover, it does not control spindle positioning through LGN/Gαi (Landin Malt et al., 2019). Interestingly, it has also been recently shown that Par3 might have an instructive role in PCP by direct binding to the core canonical PCP component Prickle3 during establishment of PCP in the Xenopus neural plate (Chuykin et al., 2018). On the other hand, Par3-dependent apicobasal polarity seems to be required to set up PCP in avian embryos (Lin and Yue, 2018). Thus, Par3/PAR-3 seems to constitute an evolutionarily conserved, context-dependent driver of PCP, either by establishing biomechanical planar polarity, relaying apicobasal polarity to planar polarity, reinforcing canonical PCP signaling, or helping to establish asymmetric localization of PCP components. Our data reveal that the early *C. elegans* embryo also requires PAR-3-dependent PCP to achieve proper signal integration and relay during axial patterning.

## Data Availability Statement

All datasets generated for this study are included in the manuscript/Supplementary Files.

## Author Contributions

PD and CP conceived the project and wrote the manuscript. PD performed all the experiments, acquired and analyzed the data. DO created the analysis software and performed the data analysis.

## Conflict of Interest

The authors declare that the research was conducted in the absence of any commercial or financial relationships that could be construed as a potential conflict of interest.
