# Supplementary material for: Planar Asymmetries in the C. elegans Embryo Emerge by Differential Retention of aPARs at Cell-Cell Contacts
Source: Front Cell Dev Biol. 2019 Sep 27;7:209. doi: 10.3389/fcell.2019.00209 (PMC6776615; doi:10.3389/fcell.2019.00209)
Supplement: Supplementary file 1 [file Image_1.pdf]

Planar asymmetries in the *C. elegans* embryo emerge by differential retention of aPARs at cell-cell contacts

Priyanka Dutta°, Devang Odedra°, Christian Pohl*

Supplementary material

Supplementary figures

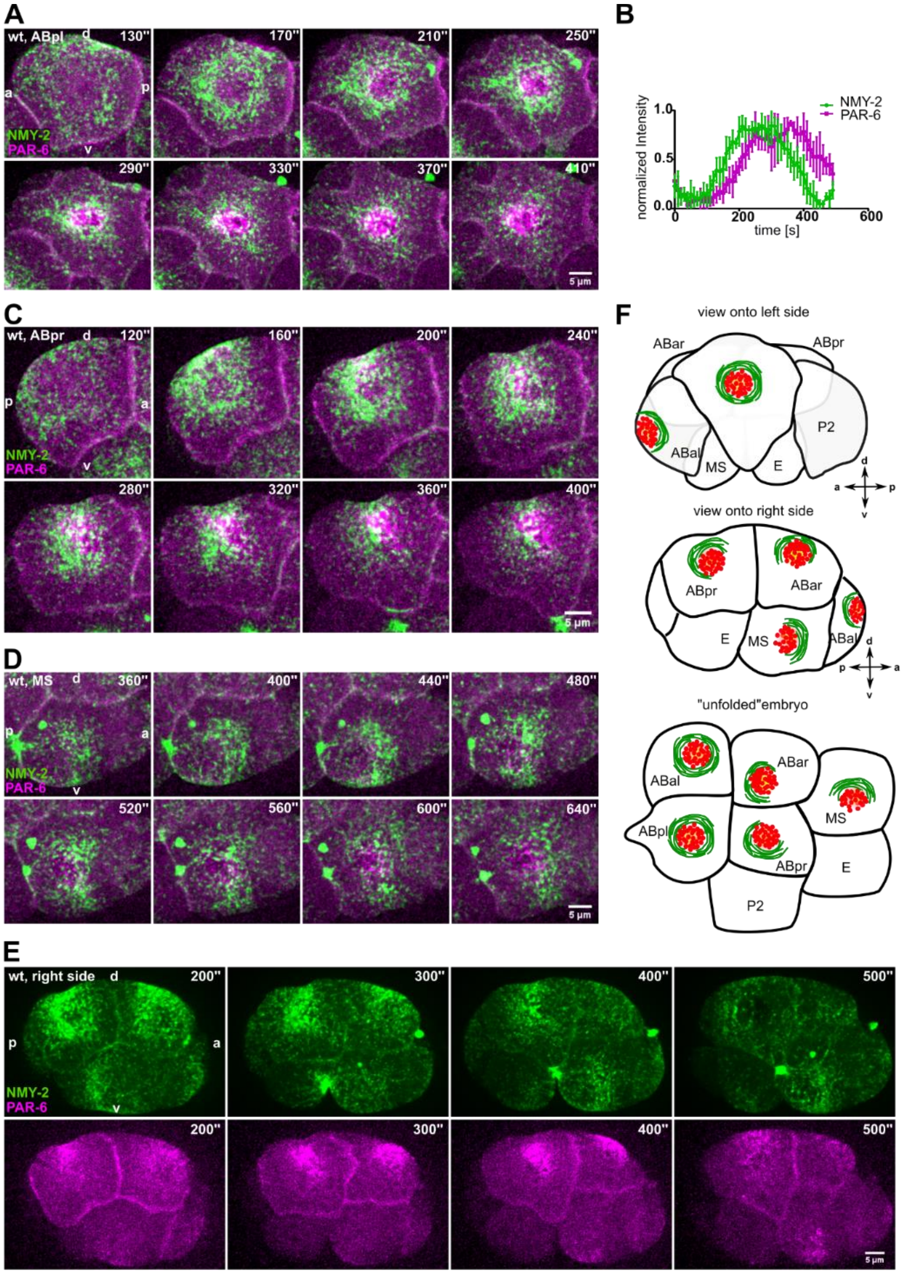

Figure S1.

(A, C-E) Representative time lapse images of apical cortical sections of ABpl (A), ABpr (C), MS (D) and a right side embryo (E) expressing NMY-2::GFP and mCherry::PAR-6. Time is with respect to the completion of ABp division. **(B)** Quantification of normalized kinetics of NMY-2::GFP and mCherry::PAR-6 along time. **(F)** Top and middle: Illustration of ABpl, ABpr and MS depicting the NMY-2-aPARs domains. Bottom: Illustration depicting how an unfolded embryo would look like. The axis directions are represented below the illustration. Scale bar = 5 µm.

targets of RNAi screen	
apr-1	*mel-11*
cdc-42	*mom-2*
csnk-1	*mom-5*
cye-1	*nmy-1*
dnc-1	*pal-1*
ect-2	*par-1*
erm-1	*pfn-1*
glp-1	*picc-1*
gpr-1	*pie-1*
lat-1	*pkc-3*
let-99	*pop-1*
lin-5	*skr-1*
lin-23	*tat-5*

Figure S2.

List of genes tested by targeted RNAi screening

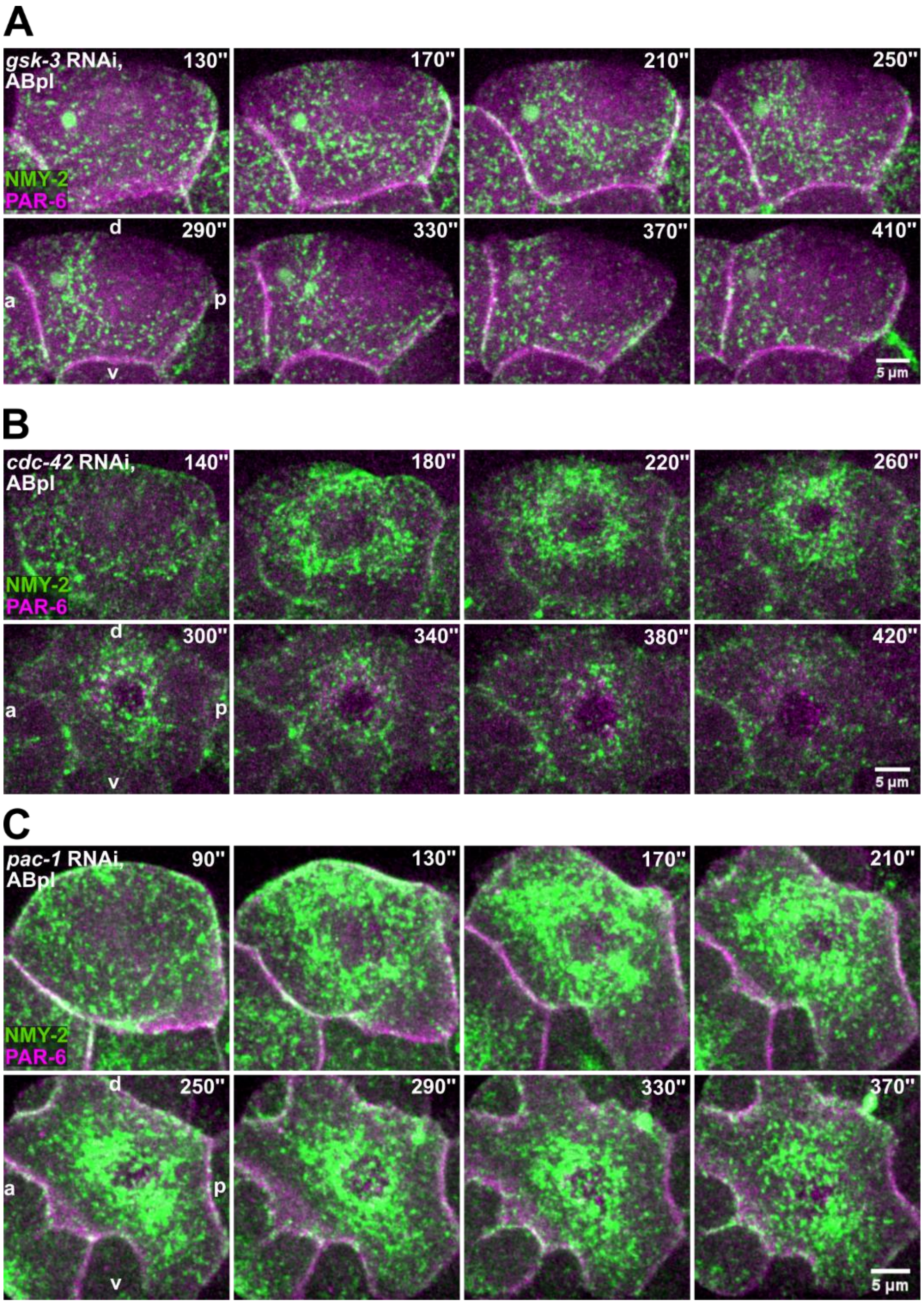

Figure S3.

(A-C) Representative time lapse images of apical cortical sections of ABpl expressing NMY-2::GFP and mCherry::PAR-6 in *gsk-3* RNAi, *cdc-42* RNAi and *pac-1(xn6)* animals. Time is with respect to the completion of ABp division. Scale bar = 5 μm.

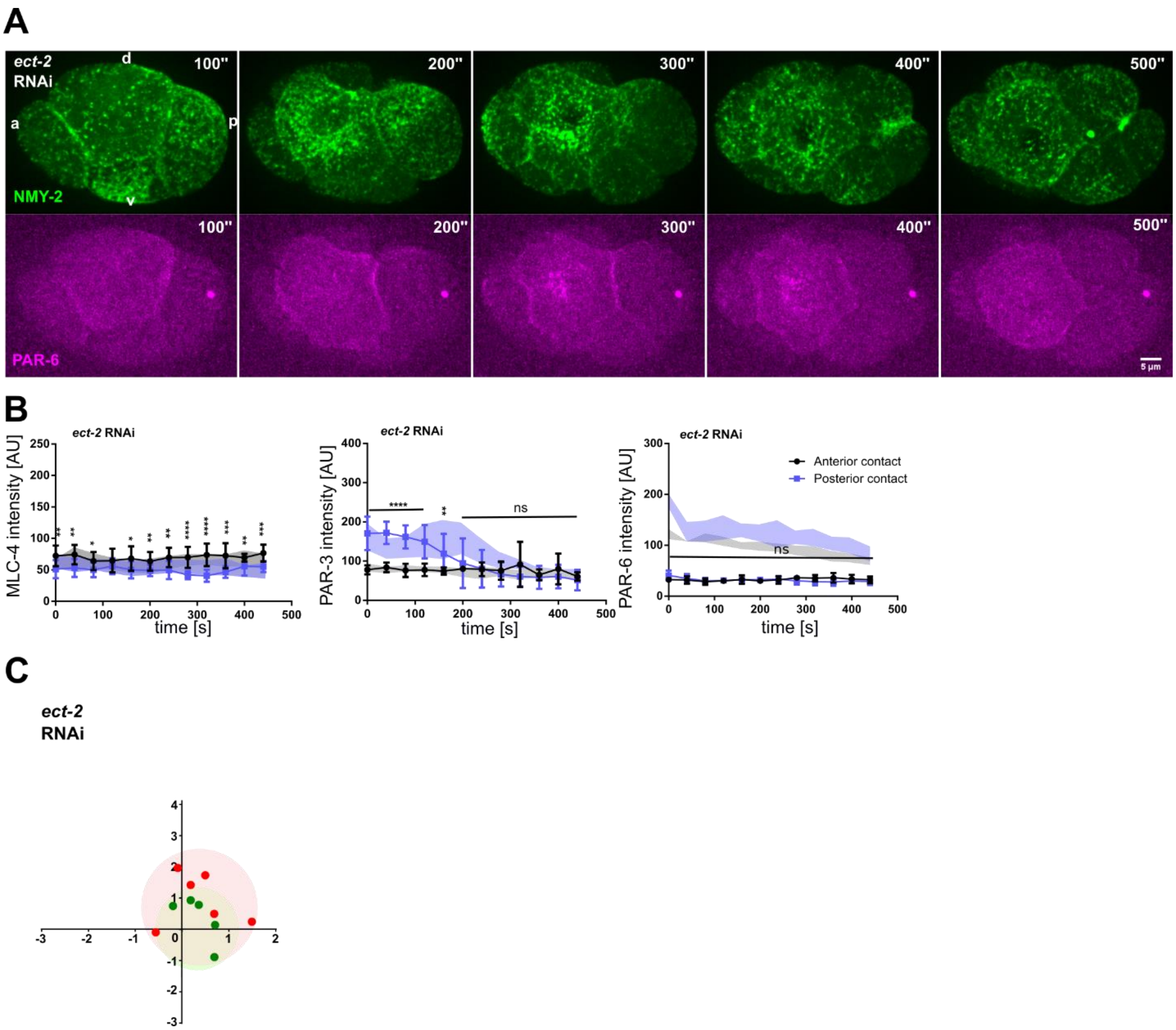

Figure S4.

(A) Representative time lapse images of apical cortical sections of ABpl expressing NMY-2::GFP and mCherry::PAR-6 in *ect-2* RNAi embryos. **(B)** Quantifications of mCherry::MLC-4, PAR-3::GFP and mCherry::PAR-6 at ABpl's anterior (ABal-ABpl) and posterior (ABpl-P2) contact measured from apical cortical sections in *ect-2* RNAi animals. **(C)** Positioning of the NMY-2-aPAR cortical domain in *ect-2* RNAi embryos with respect to the center of mass of the ABpl cell cortex which is taken as coordinate (0,0). Scale bar = 5 µm.

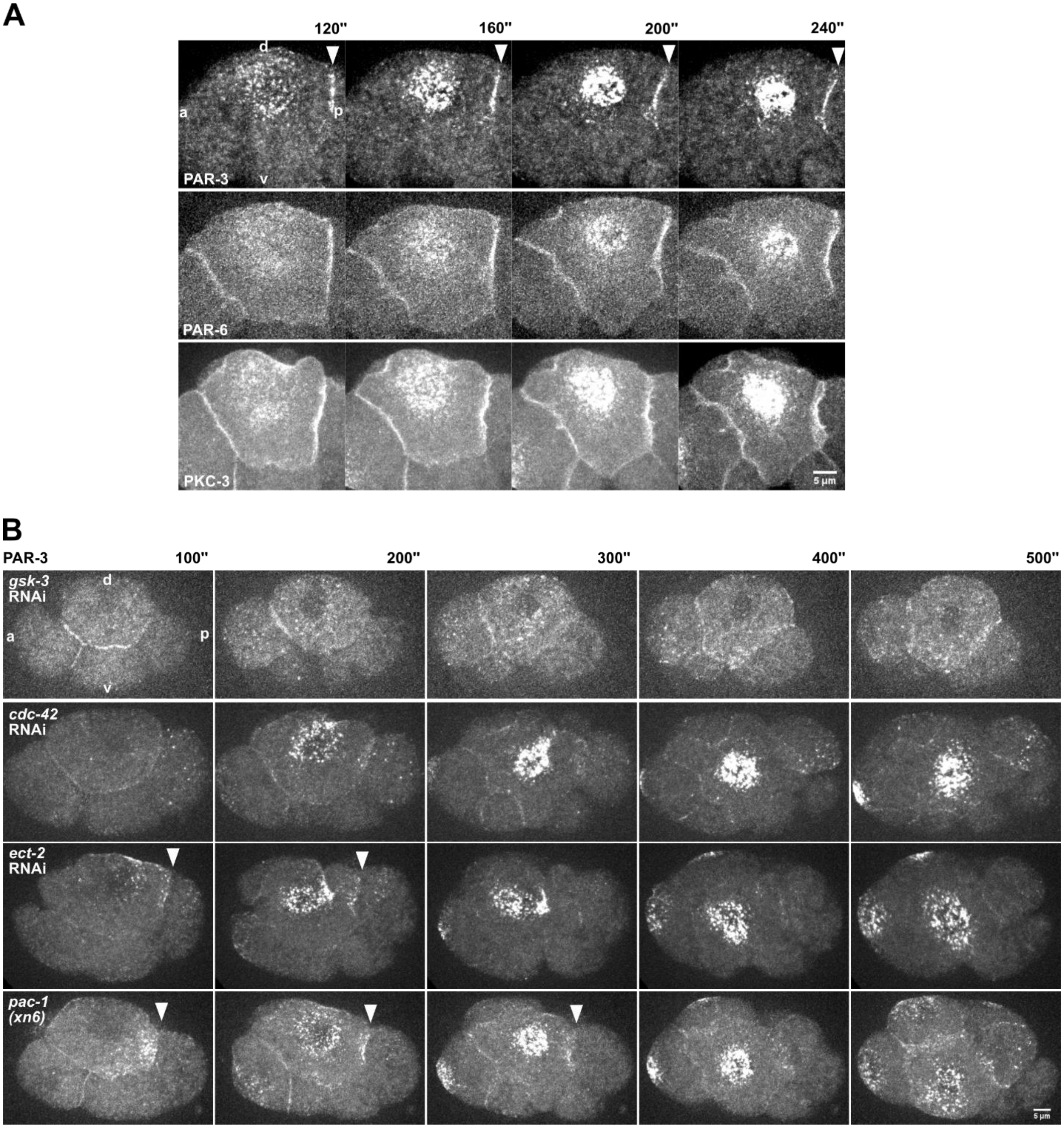

Figure S5.

(A) Representative time lapse images of ABpl apical cortical sections expressing PAR-3::GFP (top), mCherry::PAR-6 (middle) and GFP::PKC-3 (bottom). Time is with respect to the completion of ABp division. White arrowheads represent asymmetric PAR-3 localization. **(B)** Representative time lapse images of cortical sections of embryos expressing PAR-3::GFP in *gsk-3* RNAi, *cdc-42* RNAi, *ect-2* RNAi and *pac-1(xn6)*. For wt, see Figure 3. White arrowheads represent asymmetric PAR-3 localization. Scale bar = 5 µm.

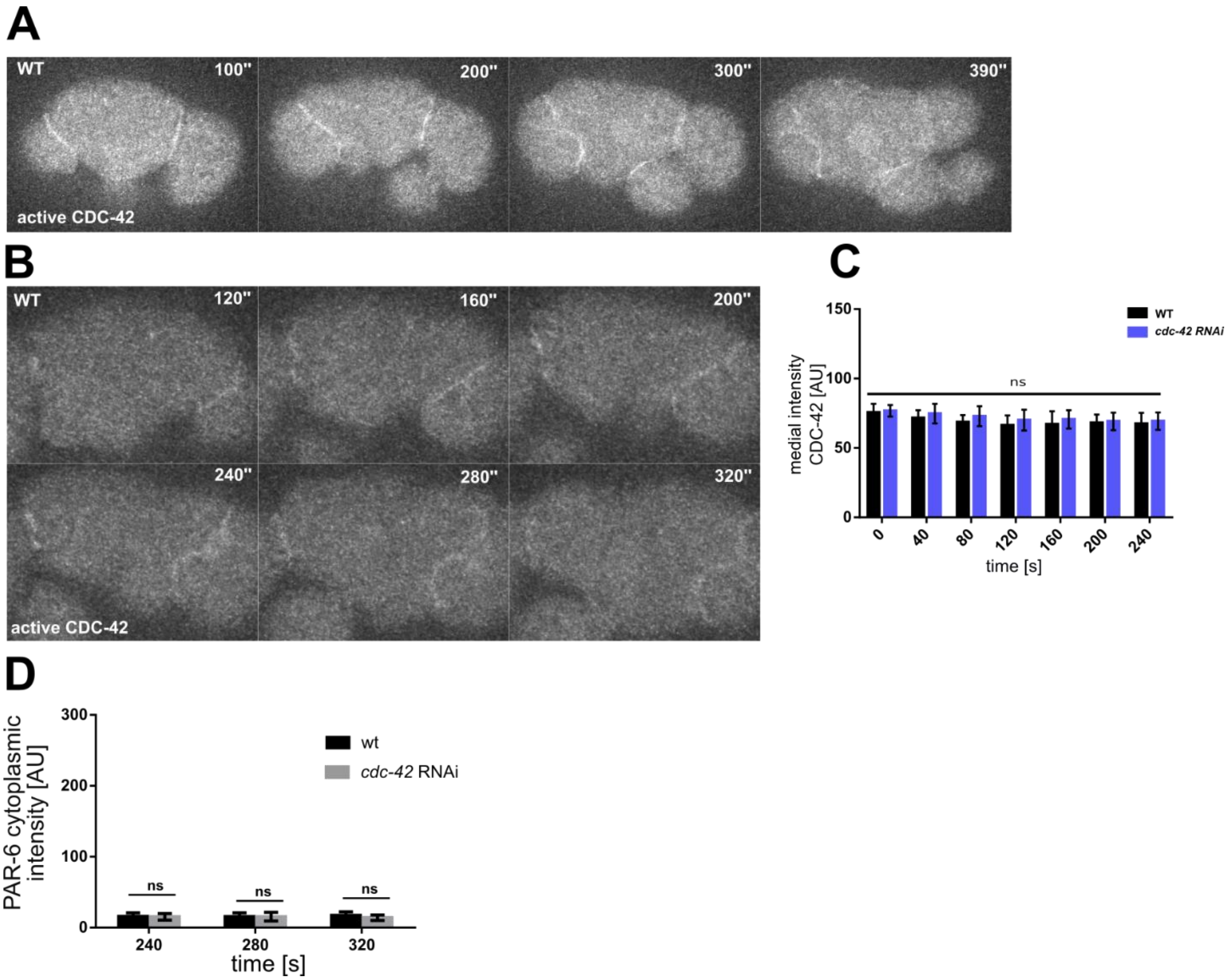

Figure S6.

(A-B) Representative time lapse images of cortical sections of an embryo expressing a GFP-tagged CRIB/G-protein binding domain of WSP-1, a sensor for activated CDC-42. Time is with respect to the completion of ABp division. Panel (B) shows magnified views of ABpl. **(C)** Comparison of medial fluorescence intensity of activated CDC-42 in wt and *cdc-42* RNAi embryos. **(D)** Quantification of mCherry::PAR-6 fluorescence intensity in the cytoplasm in wt and cdc-42 RNAi embryos (n = 5). Scale bar = 5 µm.

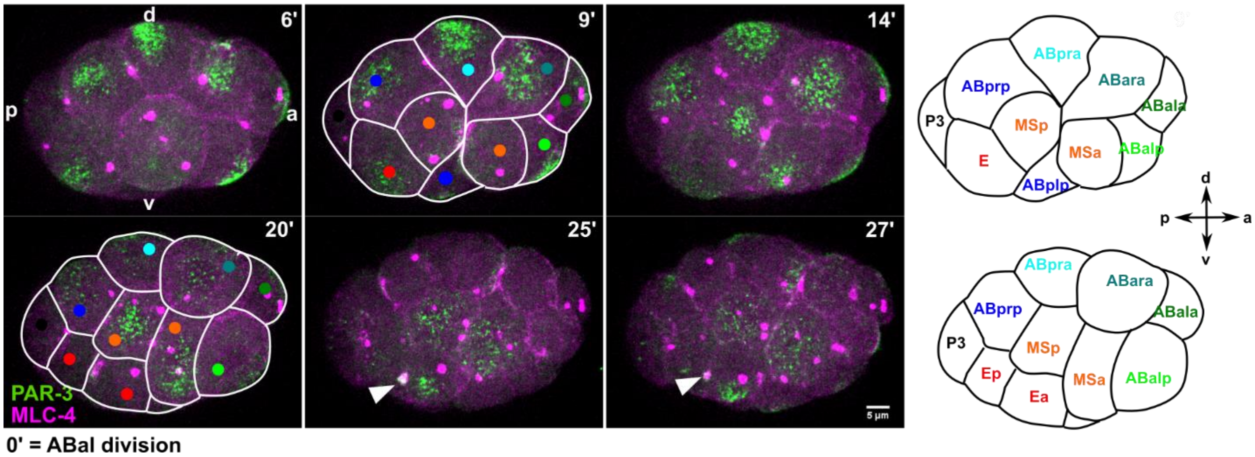

Figure S7.

Representative time lapse images of cortical sections of embryos expressing PAR-3::GFP and mCherry::MLC-4 starting at the 12 cell stage until shortly before Ea/Ep gastrulate. White outlines depict cells intercalating into the MS furrow; white arrowhead marks the colocalization of PAR-3 with the E midbody. Scale bar = 5 µm.

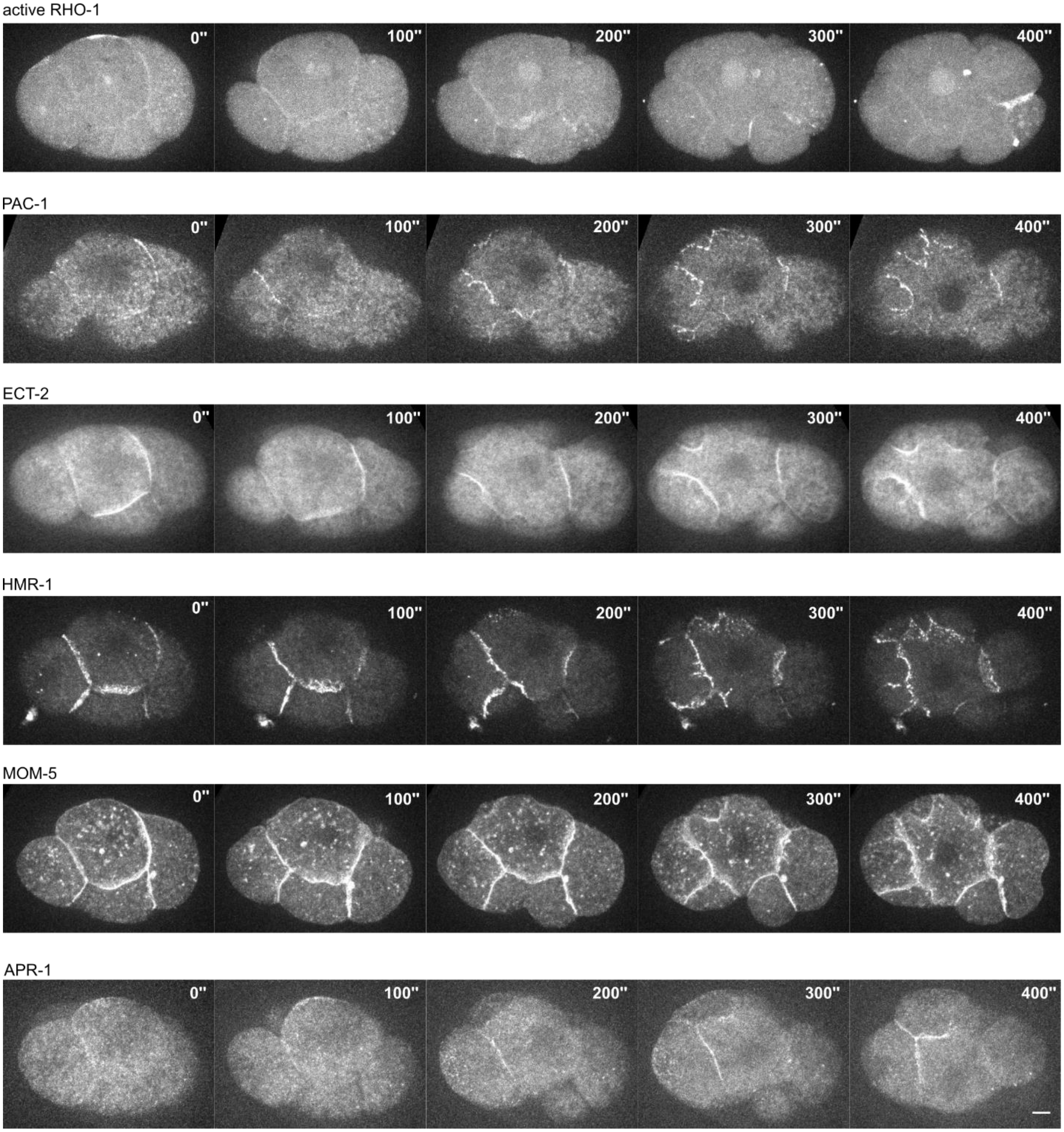

Figure S8.

Representative time lapse images of cortical sections of embryos expressing GFP::ANI-1, GFP::PAC-1, ECT-2::GFP, HMR-1::GFP, MOM-5::mNG, mNG::APR-1. Time is with respect to the completion of ABp division. Scale bar = 5 µm.

Supplementary videos

Video S1.

Representative time lapse series showing a wt embryo expressing NMY-2::GFP and mCherry::PAR-6. Time is with respect to the completion of ABp division. Shown is the left side.

Video S2.

Representative time lapse series showing a wt embryo expressing NMY-2::GFP and mCherry::PAR-6. Time is with respect to the completion of ABp division. Shown is the right side.

Video S3.

Representative time lapse series showing a *gsk-3* RNAi embryo expressing NMY-2::GFP and mCherry::PAR-6. Time is with respect to the completion of ABp division. Shown is the left side.

Video S4.

Representative time lapse series showing a *cdc-42* RNAi embryo expressing NMY-2::GFP and mCherry::PAR-6. Time is with respect to the completion of ABp division. Shown is the left side.

Video S5.

Representative time lapse series showing a *pac-1(xn6)* embryo expressing NMY-2::GFP and mCherry::PAR-6. Time is with respect to the completion of ABp division. Shown is the left side.

Video S6.

Representative time lapse series showing an *ect-2* RNAi embryo expressing NMY-2::GFP and mCherry::PAR-6. Time is with respect to the completion of ABp division. Shown is the left side.

Video S7.

Combined representative time lapse series showing wt embryos expressing mCherry::PAR-6 (left), GFP::PKC-3 (middle) and PAR-3::GFP (right). Time is with respect to the completion of ABp division. Shown is the left side of embryos.

Video S8.

Combined representative time lapse series showing wt embryos expressing PAR-3::GFP, left side of an embryos on the left and a right side on the right. Time is with respect to the completion of ABp division.

Video S9.

Combined representative time lapse series showing wt, *gsk-3* RNAi, *cdc-42* RNAi and *pac-1(xn6)* embryos expressing PAR-3::GFP. Time is with respect to the completion of ABp division. Shown is the left side of embryos.

Video S10.

Representative time lapse series showing a wt embryo expressing PAR-3::GFP starting at the 12 cell stage until shortly before Ea/Ep gastrulate. Shown is the right side.

Video S11.

Representative time lapse series showing a wt embryo expressing PAR-3::GFP starting at the 12 cell stage until shortly before C divides. Shown is the left side.

Supplementary tables

Table S1.

Precise timing of the three phases of cortical flow during chiral morphogenesis based on kinematic classification.

Table S2.

Genotypes of *C. elegans* strains used in this study.
